# ZFP36L2 is a cell cycle-regulated CCCH protein necessary for DNA lesion-induced S-phase arrest

**DOI:** 10.1242/bio.031575

**Published:** 2018-02-15

**Authors:** Aya Noguchi, Shungo Adachi, Naoto Yokota, Tomohisa Hatta, Tohru Natsume, Hiroyuki Kawahara

**Affiliations:** 1 Laboratory of Cell Biology and Biochemistry, Department of Biological Sciences, Tokyo Metropolitan University, Tokyo 192-0397, Japan; 2 Molecular Profiling Research Center for Drug Discovery, National Institute of Advanced Industrial Science and Technology (AIST), Tokyo 135-0064, Japan

**Keywords:** Cell cycle, DNA damage response, Ubiquitin, Protein degradation, CCCH-zinc finger domain, RNA-binding protein

## Abstract

ZFP36L2 promotes the destruction of AU-rich element-containing transcripts, while its regulation and functional significance in cell cycle control are scarcely identified. We show that ZFP36L2 is a cell cycle-regulated CCCH protein, the abundance of which is regulated post-translationally at the respective stages of the cell cycle. Indeed, ZFP36L2 protein was eliminated after release from M phase, and ZYG11B-based E3 ligase plays a role in its polyubiquitination in interphase. Although ZFP36L2 is dispensable for normal cell cycle progression, we found that endogenous ZFP36L2 played a key role in cisplatin-induced S-phase arrest, a process in which the suppression of G1/S cyclins is necessary. The accumulation of ZFP36L2 was stimulated under DNA replication stresses and altered interactions with a subset of RNA-binding proteins. Notably, silencing endogenous *ZFP36L2* led to impaired cell viability in the presence of cisplatin-induced DNA lesions. Thus, we propose that ZFP36L2 is a key protein that controls S-phase progression in the case of genome instability.

## Abstract (precis)

**Summary:** ZFP36L2 is a cell cycle-regulated RNA-binding protein, the abundance of which is regulated post-translationally. This protein is especially accumulated in and critical for the survival of DNA-damaged cells.

## INTRODUCTION

The expression of many cell cycle regulatory proteins is strictly controlled at the respective stages of the cell cycle (Whitfield et al., 2002). Regulation of their transcription and post-translational modification has been investigated extensively (Vodermaier, 2004; Wittenberg and Reed, 2005; Guardavaccaro and Pagano, 2006; Benanti, 2012). For example, the amounts of G1/S cyclins, peaking at G1 or S phase, are thought to be controlled mainly at the transcriptional level (Wittenberg and Reed, 2005), whereas B-type cyclins are targeted for destruction by M-phase-specific ubiquitination machinery, with their protein abundance peaking at the boundary of the metaphase to anaphase transition (Glotzer et al., 1991; King et al., 1995; Sudakin et al., 1995). The turnover of transcribed mRNAs might also be an important regulatory mechanism ensuring rapid and accurate gene expression, but its importance in cell cycle control remains to be fully elucidated.

Subsets of cell cycle proteins including cyclins are encoded by mRNAs containing AU-rich element (ARE) in their 3′-untranslated region (UTR) (Bakheet et al., 2006; Spasic et al., 2012; Mukherjee et al., 2014). ARE is a major determinant of mRNA stability, leading to rapid mRNA decay, and up to 8% of all mRNAs possess AREs (Bakheet et al., 2006). Class II-type AREs (typically consisting of the palindromic sequence 5′-UAUUUAU-3′) within the 3′-UTR are recognized by the CCCH-type zinc-finger domain family of mRNA-binding proteins with high binding affinity (Carballo et al., 1998, 2000; Lai et al., 1999, 2000; Blackshear et al., 2003; Hudson et al., 2004; Lykke-Andersen and Wagner, 2005). When CCCH-domain proteins bind to an ARE-containing mRNA, they promote its deadenylation and destruction (Hau et al., 2007; Lykke-Andersen and Wagner, 2005; Sandler et al., 2011; Schoenberg and Maquat, 2012), thereby down-regulating the translation of target transcripts.

The ZFP36 family proteins tristetraprolin (TTP) (also called TIS11 or ZFP36) and the butyrate response factors 1 and 2 (also called ZFP36L1 and ZFP36L2, respectively) are prototypical members of a family of mammalian proteins that possess two tandem CCCH-type zinc-finger domains (Varnum et al., 1991; Blackshear, 2002). Mammalian TTP was identified initially as a positive regulator for eliminating tumor necrosis factor α (TNFα) mRNA, an ARE-containing transcript, by studying the autoimmune-like phenotype of TTP knockout (KO) mice (Taylor et al., 1996; Carballo et al., 1998; Blackshear, 2002; Brooks and Blackshear, 2013). ZFP36L1 was identified in a functional genetic screen to find genes responsible for ARE-dependent mRNA decay (Stoecklin et al., 2002; Schmidlin et al., 2004). ZFP36L2, the major subject of this paper, is a less well-characterized ZFP36L1-related gene product. ZFP36L1 and ZFP36L2 share nearly identical CCCH zinc-finger domains with moderate (48%) overall amino acid sequence identity. Increasing evidence suggests that gene disruption of the ZFP36 family proteins results in unique phenotypes. For example, TTP KO mice appear normal at birth, but within 8 weeks they exhibit a systemic inflammatory phenotype that is largely due to increased TNFα secretion (Taylor et al., 1996; Lai et al., 2006). However, ZFP36L1 KO embryos die presumably due to failure of placental function *in utero*, between approximately E8 and E12, when ZFP36L1 mRNA is highly expressed in the mouse embryo (Stumpo et al., 2004). Homozygous ZFP36L2 KO mice are born at the expected Mendelian frequency, but most of them die within 2 weeks of birth with significantly decreased levels of red blood cells, white blood cells, and platelets (Stumpo et al., 2009). In addition, mice with decreased expression of an N-terminal-truncated form of ZFP36L2 exhibit disrupted early development with arrest at the two-cell embryonic stage (Ramos et al., 2004). The unique phenotypes induced by defective ZFP36L2 suggest that the function of this protein might not be completely redundant with that of ZFP36L1, TTP, or any other gene product. However, its participation in and regulatory mechanism of cell cycle control remain largely obscure.

In this study, we provide the first evidence that ZFP36L2 is a novel cell cycle-regulated CCCH-domain protein, the abundance of which is regulated post-translationally, depending on the stage of the cell cycle. ZFP36L2 is co-precipitated with polyubiquitin, and the polyubiquitination is mediated by the ZYG11B-based E3 ubiquitin ligase complex. Furthermore, the association of ZYG11B with ZFP36L2 protein is regulated in a cell cycle-dependent manner. We found that DNA replication defects accelerated the accumulation of ZFP36L2 protein, and endogenous ZFP36L2 played a key role in cisplatin (CDDP)-induced S-phase arrest, a process in which the suppression of G1/S cyclins is necessary. Thus, our approach has revealed a new class of regulatory mechanisms for a CCCH family RNA-binding protein that is required for maintaining DNA replication integrity during mammalian S-phase progression.

## RESULTS

### Cell cycle stage-specific changes in the amount of ZFP36L2 protein

ZFP36L2 belongs to a family of vertebrate RNA-binding CCCH-type zinc finger proteins and participates in the targeted degradation of ARE-containing transcripts. However, its regulation is poorly characterized. Given previous reports by our group and others that the nematode CCCH protein MOE-2/OMA-2 is susceptible to meiotic cell cycle stage-dependent protein degradation in oocytes and early embryos (Shimada et al., 2002, 2006a, b; Pellettieri et al., 2003; Shirayama et al., 2006), we investigated here whether ZFP36L2 protein is also subjected to cell cycle-dependent quantitative control in mammalian somatic cells. To estimate the amount and stability of ZFP36L2 protein irrespective of its transcriptional level, we constitutively expressed *ZFP36L2* in human cells synchronized at G1 phase (serum-free cultivation), G1/S phase (aphidicolin treatment), S phase (double-thymidine treatment), G2 phase (RO-3306 treatment), or M phase [thymidine-nocodazole treatment or the expression of destruction-box (D-box) mutated cyclin B1]. The integrity of cell cycle synchronization at the respective stages was verified by flow cytometric analysis (Fig. S1).

**Fig. 1.**
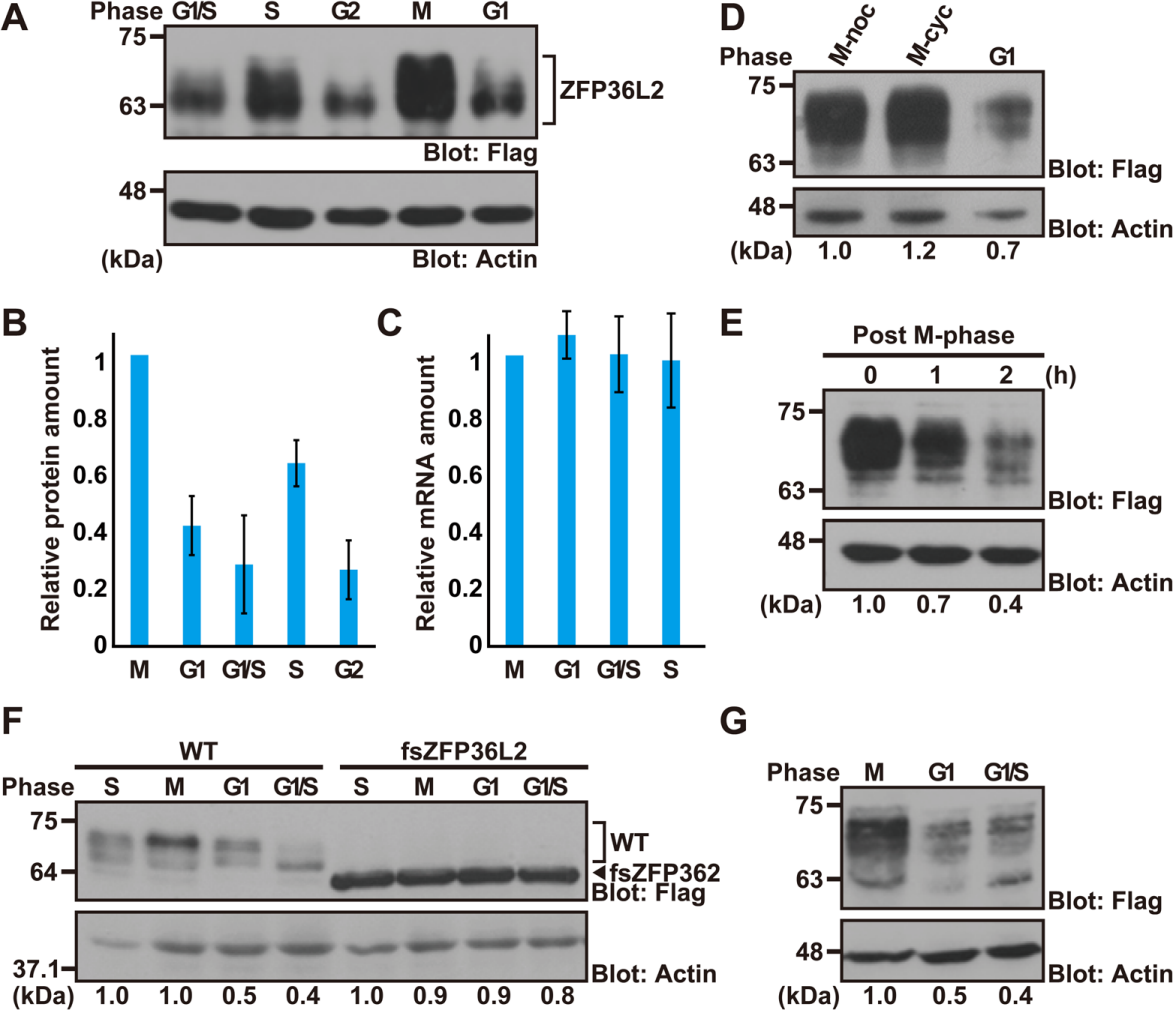
**Cell cycle stage-dependent changes in the abundance of ZFP36L2 protein.** (A) HeLa cells were transfected with an expression plasmid encoding Flag-tagged human ZFP36L2 and synchronized to each cell cycle stage: G1/S phase, early S phase, G2 phase, M phase, and G1 phase. Amounts of ZFP36L2 protein in each cell cycle stage were detected using an anti-Flag antibody. Actin was used as a loading control. Integrity of cell cycle synchronization at the respective stages was verified by flow cytometry (see also Fig. S1A). Note that WT ZFP36L2 protein can be detected as multiple (or smear) bands due to its probable post-translational modification. (B,C) Levels of ZFP36L2 protein (normalized to actin immunosignals, B) as well as its transcripts (standardized to *ACTB* mRNA levels, C) were quantified at various cell cycle stages. Semi-quantitative RT-PCR analysis supported the constant expression of the *ZFP36L2* transcript derived from the pCI-neo-based mammalian expression vector irrespective of the cell cycle arrested stage. The graph shows the quantification of anti-Flag immunosignals normalized to the actin signal at each stage, and represents the mean±s.d. calculated from at least three independent biological replicates (*n*=3). (D) All experiments were performed as in A, except for arresting the cells at M phase by over-expressing the D-box mutant form of non-degradable mitotic cyclin B1 (indicated as M-cyc). M-noc indicates M-phase-arrested cells by thymidine-nocodazole-block. (E) HeLa cells transfected with Flag-ZFP36L2 were arrested in M phase by thymidine-nocodazole treatment, then released by washing with normal medium, and chased during the course of synchronized cell cycle progression. Cells were harvested at the indicated time points and immunoblotted with an anti-Flag antibody to quantify the change in Flag-ZFP36KL2 levels. The time when nocodazole-containing mediums was replaced by normal medium was defined as time zero. (F) Wild-type ZFP36L2 (WT) as well as its frameshift mutant (fsZFP36L2) were expressed in HeLa cells under identical experimental conditions as in A. (G) Flag-tagged ZFP36L2 was expressed in HCT116 cells and probed with an anti-Flag antibody under identical experimental conditions as in A. Integrity of cell cycle synchronization at the respective stages was verified by flow cytometry (see also Fig. S1B). All experiments shown in this figure were replicated independently at least three times. Densitometry quantifications of ZFP36L2 immunoblot signals relative to loading control (actin) are indicated under the figure.

We found that wild-type (WT) ZFP36L2 protein was greatly down-regulated in G1-phase-arrested HeLa cells compared to M-phase-arrested cells (Fig. 1A,B,D). Furthermore, we found that ZFP36L2 protein was down-regulated rapidly after release from M-phase arrest by washing out nocodazole (Fig. 1E). Such a post-mitotic down-regulation of ZFP36L2 protein could not be accounted for by differences in transcriptional efficiency, since quantitative RT-PCR analysis indicated that there was no change in the amount of *Flag-ZFP36L2* transcripts at the respective stages of the cell cycle, in contrast to the changes in its protein level (Fig. 1B,C). Furthermore, a frameshift mutation at residue 145 of ZFP36L2 (designated as fsZFP36L2, encoding a 59-kDa protein) completely abolished its cell cycle dependency under identical experimental conditions (Fig. 1F), suggesting that differences in translational efficiency (and any other pre-translational differences) at the respective cell cycle stages could not account for the cell cycle dependency of WT ZFP36L2 protein. Collectively, the unique cell cycle behavior of WT ZFP36L2 protein must be determined by a post-translational mechanism and is governed by its own primary sequence. In addition, we confirmed that ZFP36L2 protein fluctuated during the cell cycle, not only in HeLa cells (Fig. 1A,D,E) but also in the near-diploid human colorectal cancer cell line HCT116 (Fig. 1G; Fig. S1B), by greatly down-regulating its protein level at the post-mitotic stages. These observations imply that ZFP36L2 is a novel mammalian CCCH-type zinc finger protein whose abundance could be regulated post-translationally during the respective stages of the cell division cycle.

### Ubiquitin-dependent machinery supports the down-regulation of ZFP36L2 protein in post-mitotic cells

**Fig. 2.**
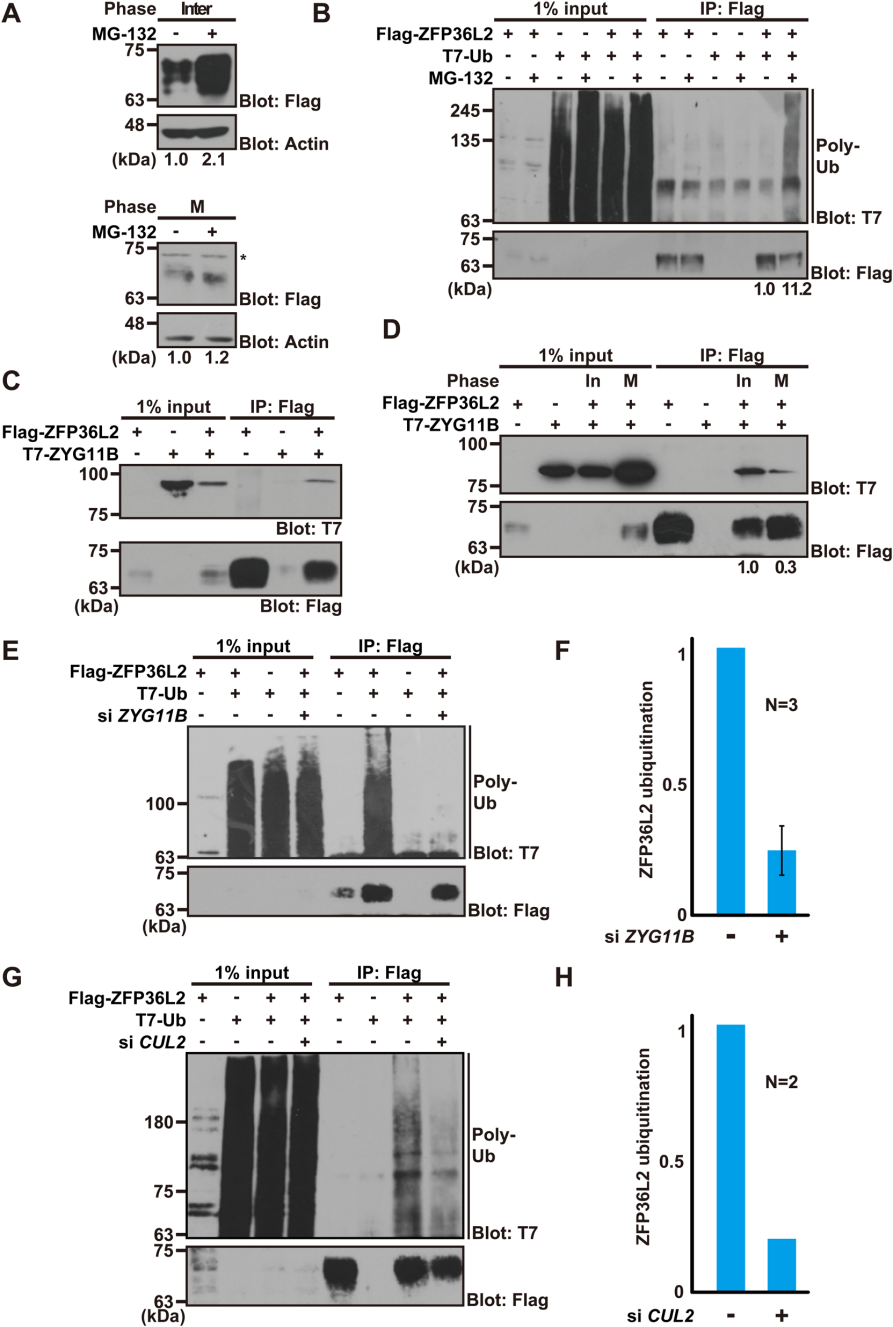
**ZYG11B-based E3 ligase supports co-precipitation of polyubiquitin with ZFP36L2.** (A) ZFP36L2 is down-regulated in interphase cells by protein degradation. HeLa cells expressing Flag-tagged ZFP36L2 were synchronized either at M phase (thymidine-nocodazole block) or interphase (serum-free cultivation), and then treated for an additional 4 h with (+) or without (−) 10 µM MG-132. Densitometry quantification of Flag immunoblot signals relative to loading control is shown below each lane. (B) Co-immunoprecipitation (IP) experiments to detect polyubiquitin association of ZFP36L2. Flag-ZFP36L2 and T7-tagged ubiquitin (T7-Ub) were co-expressed in HeLa cells, and the cells were treated with (+) or without (−) 10 µM MG-132 for 4 h. Anti-Flag immunoprecipitates from the protein lysates were probed with an anti-T7 antibody to detect polyubiquitin co-precipitation of ZFP36L2. Note that ZFP36L2 loading was adjusted. Densitometry quantification of T7 immunoblot signals relative to Flag signals is shown below each lane. (C) ZFP36L2 physically interacts with ZYG11B protein. Flag-tagged ZFP36L2 was co-expressed in HeLa cells with T7-tagged ZYG11B. An anti-Flag M2 antibody was used for immunoprecipitation. (D) Interaction of ZFP36L2 with ZYG11B was enhanced in interphase cells. HeLa cells expressing Flag-tagged ZFP36L2 and T7-ZYG11B were harvested either at interphase (In) or M phase (M), and then ZFP36L2 was immunoprecipitated using an anti-Flag M2 antibody and probed with an anti-T7 antibody. Densitometry quantification of T7 immunoblot signals relative to Flag signals is shown below each lane. (E-H) *ZYG11B* and *CUL2* knockdown weakened the co-precipitation of polyubiquitin with ZFP36L2 protein. Flag-tagged ZFP36L2 and T7-Ub were expressed in siRNA-treated HCT116 cells with MG-132 (E,G). Flag precipitates were probed with an anti-T7 antibody to detect the co-precipitation of polyubiquitin with ZFP36L2. Graphs indicate the quantified data of the polyubiquitin blot signals that were co-immunoprecipitated with ZFP36L2 protein from *ZYG11B* knockdown cells (F) and *CUL2* knockdown cells (H). *ZYG11B* knockdown experiments were replicated independently three times, and *CUL2* knockdown experiments were replicated twice. The efficacy of *ZYG11B* and *CUL2* siRNA knockdown was verified by western blot analysis (see also Fig. S3A,B).

Given that significant differences in Flag-ZFP36L2 protein expression were observed during the cell cycle, irrespective of its constant mRNA levels (Fig. 1B,C), we speculated that the post-mitotic down-regulation of ZFP36L2 protein (Fig. 1E) might be mediated by enhanced protein degradation. Thus, we examined whether inhibition of intracellular proteolysis restored ZFP36L2 accumulation in interphase cells. When the protease inhibitor MG-132 was added to interphase cells for 4 h, ZFP36L2 protein accumulated greatly (Fig. 2A). In contrast, M-phase-arrested cells showed little sensitivity to MG-132 treatment with respect to the protein abundance of ZFP36L2 (Fig. 2A). These observations suggest that ZFP36L2 is down-regulated in interphase cells by protein degradation.

Polyubiquitin modification is a key process for intracellular protein destruction (Benanti, 2012; Suzuki and Kawahara, 2016). Therefore, we next investigated whether ZFP36L2 is polyubiquitinated. We found that a polyubiquitin moiety co-precipitated efficiently with ZFP36L2 only in the presence of MG-132 (Fig. 2B), suggesting that ZFP36L2 is subjected to ubiquitin-dependent protein degradation in interphase cells.

It has been shown that the destruction of B-type cyclins at the exit of mitosis is executed redundantly by the anaphase-promoting complex (APC)/cyclosome (APC/C)- and/or ZYG11B-dependent polyubiquitination pathway (King et al., 1995; Sudakin et al., 1995; Harper et al., 2002; Guardavaccaro and Pagano, 2006; Benanti, 2012, Balachandran et al., 2016). D-box-mediated ubiquitination machinery is activated through late M phase to G1/S phase (King et al., 1995; Sudakin et al., 1995), while the nocodazole-induced spindle assembly checkpoint suppresses APC/C activation. Similar to the case in cyclins, ZFP36L2 protein seemed to be down-regulated at the post-mitotic stages (Fig. 1), at which time (late M phase to G1 phase) the cyclin degradation machinery might be activated (Harper et al., 2002). Inspection of the ZFP36L2 amino acid sequence revealed that there are conserved D-box-like (^239^RdaLhlgfp, ^251^RpkLhhslS) and KEN-box-like (^115^KENkfrD) sequences (Fig. S2A,B), both of which were seemingly putative APC/C recognition motifs for ubiquitination (Glotzer et al., 1991; Pfleger and Kirschner, 2000). To examine whether these sites contribute to the instability and cell cycle dependency of ZFP36L2, we mutated several core residues of these sequences. As shown in Fig. S2C, disruption of neither sequence significantly influenced the post-mitotic elimination of ZFP36L2 protein. These observations suggest that APC/C might not be the sole contributor to the cell cycle-dependent elimination of this protein at early interphase.

ZYG-11, the nematode homolog of human ZYG11B, was identified originally as a substrate-recognition module of the CUL-2-based E3 ubiquitin ligase complex in *Caenorhabditis elegans* (Kemphues et al., 1986; Sonneville and Gönczy, 2004; Liu et al., 2004; Vasudevan et al., 2007), and is required for the proteasome-mediated degradation of OMA-family CCCH-type zinc finger proteins during nematode meiotic cell cycle progression (Shimada et al., 2002, 2006a; DeRenzo et al., 2003; Shirayama et al., 2006). Mammalian ZYG11B was reported recently to interact physically with B-type cyclins and to function parallel to APC/C for their degradation (Liu et al., 2004; Sonneville and Gönczy, 2004; Balachandran et al., 2016). If human ZYG11B plays a role in ZFP36L2 ubiquitination, we would expect these proteins to interact, as in the case of cyclin B substrates. Therefore, we examined whether ZYG11B could be co-immunoprecipitated with ZFP36L2. This experiment revealed the reproducible association of these two proteins (Fig. 2C). Importantly, the predominant association of ZYG11B protein with ZFP36L2 was obvious in interphase cells rather than in M-phase-arrested cells (Fig. 2D), even though far more bait protein (Flag-ZFP36L2) accumulated in M-phase cells than in interphase cells (see input lanes of the Flag blot in Fig. 2D). These observations suggest that the association between ZYG11B and ZFP36L2 protein is modulated in a cell cycle-dependent manner.

To address whether ZYG11B contributes to the polyubiquitin modification of ZFP36L2 protein, we performed siRNA-mediated knockdown of *ZYG11B* expression (Fig. S3A). As shown, *ZYG11B* siRNA weakened the co-precipitation of polyubiquitin with ZFP36L2 protein (Fig. 2E,F), not only in HeLa cells but also in HCT116 cells. Furthermore, similar knockdown effects were also observed in the case of the ZYG11B-associated Cullin-family protein gene *CUL2* (Fig. 2G,H; Fig. S3B). These results suggest that ZYG11B-CUL2-based E3 complexes play a role in the co-precipitation of polyubiquitin with ZFP36L2 protein, although there might be some redundancy with APC/C ubiquitin ligase (Fig. S2) and other cellular protein degradation machineries.

Nocodazole-induced M-phase arrest is known to be associated with the spindle assembly checkpoint (Blajeski et al., 2002; Jia et al., 2013). Therefore, we initially wondered whether the accumulation of ZFP36L2 protein in M-phase-arrested cells might have resulted from activation of this checkpoint. We concluded that this was not likely for M phase, since the forced expression of the non-degradable cyclin B1 protein (an alternative method for inducing M-phase arrest without activating the spindle assembly checkpoint) also resulted in the accumulation of ZFP36L2 protein (M-cyc in Fig. 1D), similar to nocodazole-induced M-phase-arrest cells (M-noc in Fig. 1D). Furthermore, we clearly showed that ZFP36L2 protein was increased drastically within 4 h of release from G2 arrest (by washing out RO-3306) during the course of non-arrested cell cycle progression (Fig. S4A,B). These observations exclude the hypothesis that the spindle assembly checkpoint might be responsible for the M-phase-specific accumulation of ZFP36L2 protein. The M-phase-specific role(s) of ZFP36L2, if any, have not been identified at present.

### C-terminal region of ZFP36L2 is critical for its instability

**Fig. 3.**
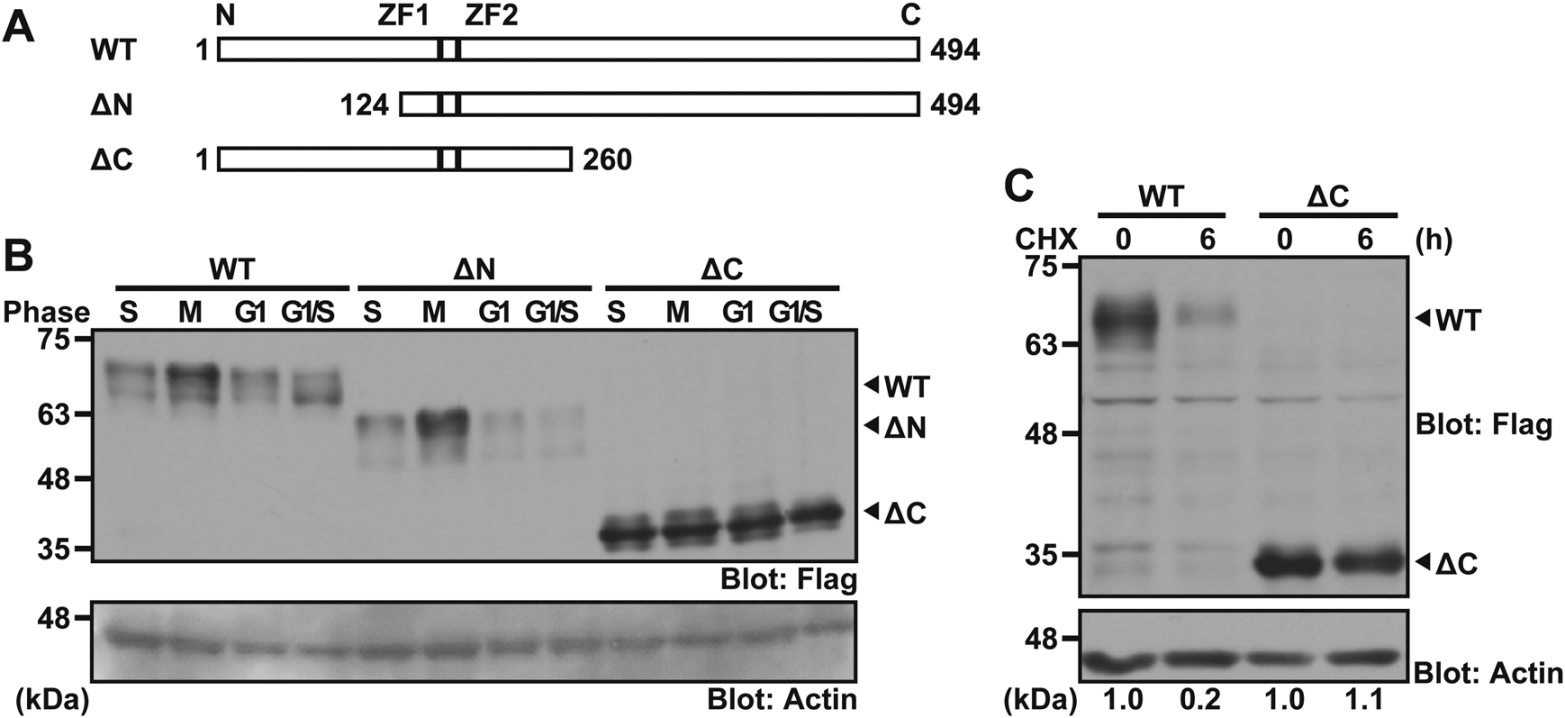
**The C-terminal region of ZFP36L2 determines its stability.** (A) Schematic representation of the deletion mutants used in this study. ZFP36L2 contains two copies of the conserved CCCH-type zinc-finger domain, designated as ZF1 and ZF2. (B) Deletion analysis to identify the region required for the cell cycle dependency of ZFP36L2 protein. The WT form of Flag-tagged ZFP36L2 and its truncated derivatives (ΔN and ΔC) were expressed in HeLa cells as in Fig. 1A. Actin was used as a loading control. (C) The C-terminal region of ZFP36L2 is essential for its instability. HeLa cells were transfected with the WT or ΔC form of Flag-tagged ZFP36L2. At 24 h after transfection, translation was blocked with 20 µg/ml cycloheximide (CHX). The cells were harvested at the indicated times after CHX addition and blotted with an anti-Flag antibody. All experiments shown in this figure were replicated at least three times. Fold changes of ZFP36L2 immunoblot signals (Flag/actin) relative to time zero (WT) is indicated under the figure.

To investigate further the region required for its cell cycle dependency, we prepared a series of truncated mutants of ZFP36L2, and investigated whether removal of the N-terminal or C-terminal region from ZFP36L2 protein modified its susceptibility to the G1/S-phase-specific decrease in its level. Two truncated fragments, ΔC (encoding amino acids 1-260) and ΔN (amino acids 124-494), as well as WT ZFP36L2 proteins were tested as substrates for cell cycle dependency *in vivo* (Fig. 3A). The results clearly showed that the deletion of the C-terminal 234 amino acid residues (i.e. ΔC) from full-length ZFP36L2 was sufficient to abolish its cell cycle dependency (Fig. 3B), while a fragment containing the C-terminal region (ΔN) was found to fluctuate during the cell cycle, similar to the case of WT protein (Fig. 3B). These results suggest that the C-terminal region of ZFP36L2 plays an essential role in the cell cycle dependency of this protein and support our findings with the frame-shift mutation (Fig. 1F).

Since the cell cycle dependency of ZFP36L2 was shown to be linked with enhanced protein degradation in the G1/S phase (Fig. 2A), we wanted to know whether WT ZFP36L2 might be more unstable than ΔC ZFP36L2. A cycloheximide (CHX)-chase experiment showed that WT ZFP36L2 was degraded rapidly with a half-life of <2 h, while the ΔC-mutant was barely degraded within 6 h of CHX addition (Fig. 3C). These results suggest that the C-terminal stretch of ZFP36L2 contains an element that determines the instability and cell cycle dependency of this protein.

### Architecture of the ZFP36L2 complex is modulated during the cell cycle

**Fig. 4.**
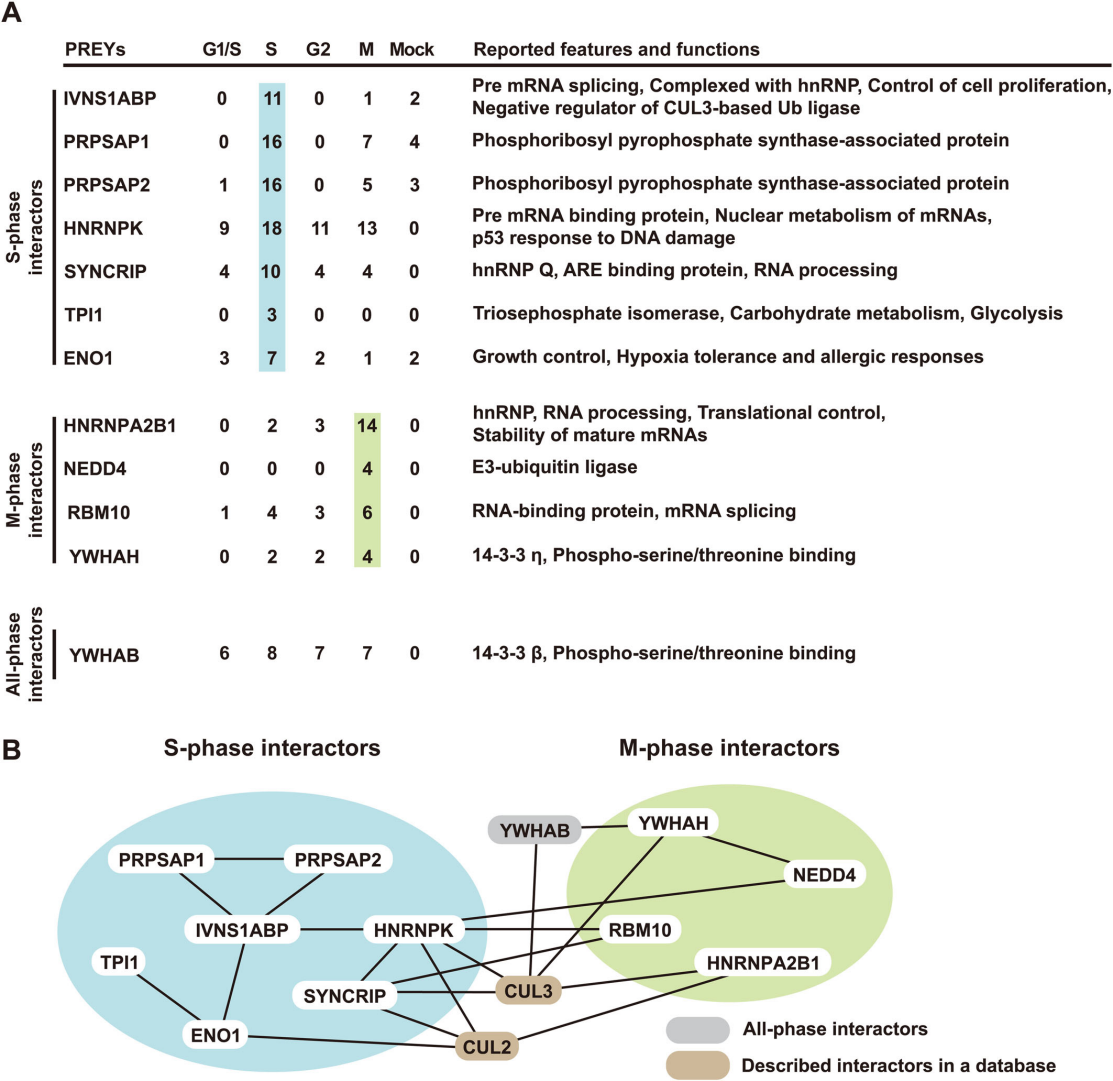
**LC/MS/MS-based analysis reveals ZFP36L2-binding proteins are specifically associated in S phase and M phase cells.** (A) N-terminal Flag-tagged ZFP36L2 was expressed in HeLa cells, and the cells were then synchronized at the G1/S, S, G2 and M phases, respectively. Flag immunoprecipitates from each cell extract were subjected to LC/MS/MS analyses. Numbers of unique hit peptides derived from ZFP36L2-interactors (gene nomenclature) are listed. Proteins that were identified in at least three independent precipitation trials by two or more peptides with a peptide expectation value of *P*<0.05 were considered reliable identifications. Immunoprecipitates from cells that were transfected with the Flag-tagged empty vector were used as negative controls (mock negative control). The complete list of ZFP36L2-interacting proteins at the respective cell cycle stages is provided in Table S1. This experiment was repeated twice. (B) The protein interaction network of the ZFP36L2-associated proteins, which were determined by our LC/MS/MS analysis as in A and by the BioGRID interaction database. Results suggest the possible cell cycle stage-specific complex formation of human ZFP36L2 in either S-phase- or M-phase-arrested cells. Note that we did not see any G1-/G2-phase-specific ZFP36L2 interactors in this analysis.

To obtain further insight into the cell cycle dependency of ZFP36L2 protein in living cells, we transfected synchronized HeLa cells with Flag-tagged ZFP36L2, and its immunoprecipitates were analysed by liquid chromatography-tandem mass spectrometry (LC/MS/MS). Our MS analysis revealed that the frequency of hit peptides derived from several ZFP36L2-associated cell endogenous proteins was changed at the respective stages of the cell cycle (Fig. 4A; Table S1). For example, a dominant association with ZFP36L2 in DNA replication-defective S-phase-arrested cells was identified for the BTB/POZ-Kelch domain protein IVNS1ABP, its binding protein PRPSAP1/2, cell cycle-linked KH domain protein hnRNP-K, and cytoplasmic RNA-binding protein SYNCRIP, whereas increased binding in nocodazole-arrested M-phase cells was observed for several proteins including the ALS-causative RNA-binding protein hnRNP-A2B1 and phospho-specific adaptor protein YWHAH (14-3-3η). In addition, our co-immunoprecipitation analysis provided direct evidence of the physical associations of IVNS1ABP, hnRNP-K, and hnRNP-A2B1 with ZFP36L2 protein, respectively. Interestingly, the BioGRID protein interaction database (Chatr-Aryamontri et al., 2017) suggested that many of these cell cycle stage-dependent ZFP36L2-interacting proteins identified by our LC/MS/MS analyses associate with each other (Fig. 4B). These findings suggest that ZFP36L2 exists as a component of RNA-binding protein complexes whose architecture might be altered at the respective stages of the cell cycle.

### Silencing of endogenous ZFP36L2 causes the up-regulation of S-phase cyclins

Some CCCH-type zinc finger proteins are known to regulate mRNA stability and expression (Lai et al., 1999; Hudson et al., 2004). For example, the transcript of *TNFα* is stabilized and results in the overproduction of its product in TTP KO mice (Carballo et al., 1998; Lai et al., 1999, 2006), and several M-phase ARE transcripts are regulated directly by TTP (Marderosian et al., 2006; Horner et al., 2009; Kim et al., 2012). Given that ZFP36L2 protein is greatly down-regulated in G1/S-phase cells (Fig. 1A,D,E,G) and that the excess expression of ZFP36L2 in HeLa cells reportedly causes a delay in S-phase progression (Iwanaga et al., 2011), the possibility emerged that ZFP36L2 might have critical roles in controlling cell cycle-related ARE transcripts. Accordingly, we next tried to identify the target transcripts of ZFP36L2, in particular those up-regulated in S-phase cells.

**Fig. 5.**
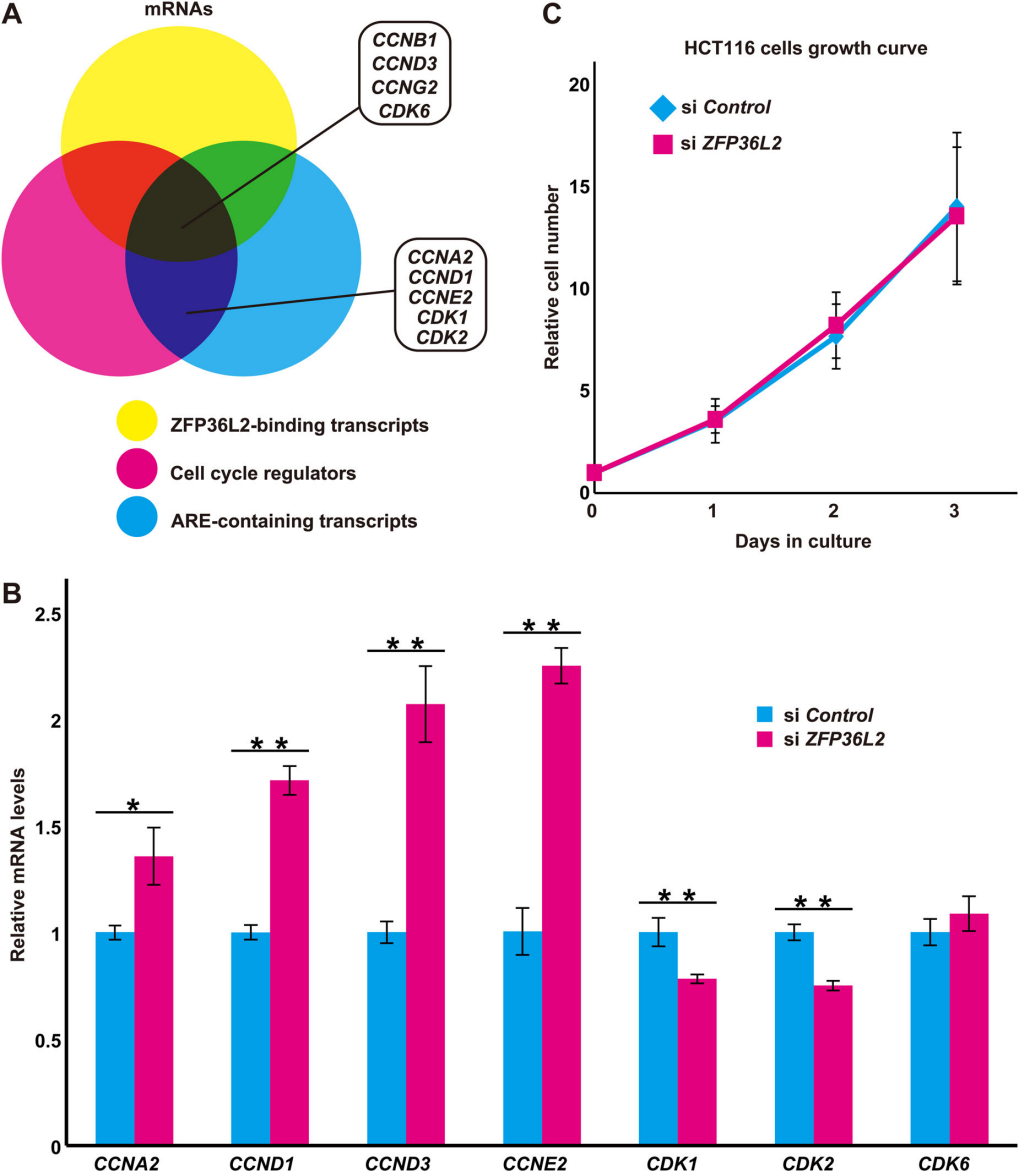
**ZFP36L2 suppresses the G1/S cyclin expressions.** (A) Venn diagram of cell cycle-related transcripts (shown as magenta) showing the overlap between mRNAs that were bound to ZFP36L2 (Zhang et al., 2013; shown as yellow) and mRNAs with AREs in their 3′-UTRs (Bakheet et al., 2006; shown as cyan). (B) Results of quantitative real-time RT-PCR analyses for potential targets of ZFP36L2 in *ZFP36L2* knockdown HCT116 cells. Knockdown of endogenous *ZFP36L2* stimulates the expression of G1/S cyclin transcripts, including cyclin A2 (*CCNA2*), cyclin D1 (*CCND1*), cyclin D3 (*CCND3*), and cyclin E2 (*CCNE2*), while the expression of CDK genes was not affected. Efficacy of *ZFP36L2* knockdown was verified by anti-Flag-ZFP36L2 immunoblot analysis (see also Fig. S3C). Data represent mean±s.d. calculated from three independent biological replicates. *P<0.05 and **P<0.01 compared with control siRNA cells. (C) Knockdown of *ZFP36L2* expression does not affect the normal proliferation of HCT116 cells. Growth curves of HCT116 cells transfected with *ZFP36L2* siRNA or control siRNA. Efficacy of *ZFP36L2* knockdown was verified by anti-Flag-ZFP36L2 immunoblot analysis (see also Fig. S3C). Data represent mean±s.d. calculated from at least three independent biological replicates.

Recently, more than 1000 transcripts that associate with ZFP36L1/ZFP36L2 proteins in hematopoietic cells were reported (Zhang et al., 2013; Galloway et al., 2016; Vogel et al., 2016). These findings, as well as our inspection of the presence of potential AREs in their 3′-UTRs and known involvement in cell cycle control prompted us to investigate whether these cell cycle-linked ARE transcripts are indeed affected by ZFP36L2 (Fig. 5A). Thus, we analyzed the mRNA levels of nine representative candidates (indicated in Fig. 5A) in HCT116 cells transfected with control siRNA or siRNA against *ZFP36L2*. As shown in Fig. 5B, we found that silencing endogenous *ZFP36L2* led to a significant increase of G1/S cyclin family mRNA levels in HCT116 cells. For example, our quantitative real-time RT-PCR analyses suggested that endogenous mRNA levels of cyclin D1 (*CCND1*), cyclin D3 (*CCND3*), cyclin E2 (*CCNE2*) and cyclin A2 (*CCNA2*) were increased in *ZFP36L2* knockdown cells by 1.7-, 2.1-, 2.3-, and 1.4-fold, respectively, compared with control knockdown cells (Fig. 5B; see also Fig. S5). In contrast, there was no significant up-regulation of the level of Cyclin dependent kinase 6 (*CDK6*) transcripts, and in the cases of *CDK1* and *CDK2*, depletion of *ZFP36L2* caused a down-regulation of their transcripts (Fig. 5B), even though all of them possess potential AREs within their 3′-UTRs. The efficacy of *ZFP36L2* knockdown by three independent siRNA duplexes was verified by western blot experiments (Fig. S3C). Since the expression of G1/S cyclins is known to have an essential role in S-phase progression (Hengstschläger et al., 1999), we examined the effects of *ZFP36L2* depletion on cell proliferation. Unexpectedly, silencing endogenous *ZFP36L2* expression in HCT116 cells did not accelerate (or delay) their proliferation (Fig. 5C). The average doubling time of *ZFP36L2* knockdown cells was 18.9 h, while that of control siRNA cells was 19.1 h. These observations suggest that ZFP36L2 is dispensable for normal cell cycle progression in HCT116 cells.

### ZFP36L2 is essential for DNA damage-induced S-phase arrest

**Fig. 6.**
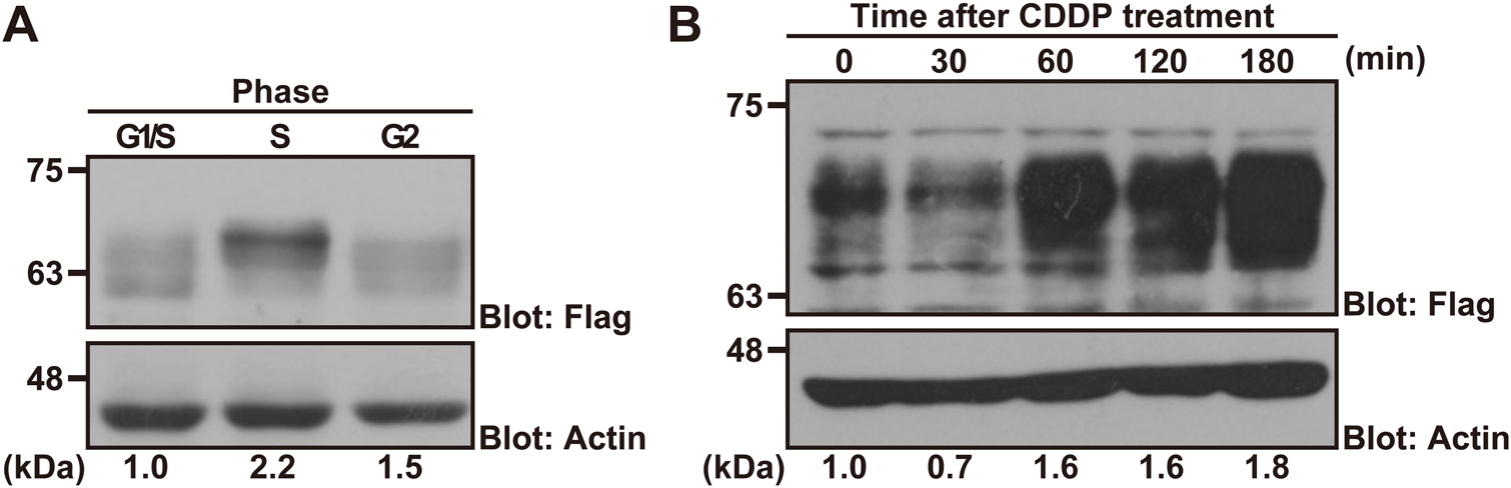
**DNA replication stresses stimulate ZFP36L2 protein accumulation.** (A) HeLa cells that were transfected with Flag-tagged ZFP36L2 were arrested either at the S-phase (induced by double-thymidine block), G1/S-phase (induced by aphidicolin), or G2-phase (induced by RO-3306). ZFP36L2 protein at the respective stages was detected by anti-Flag immunoblot analysis. Densitometry quantification of Flag immunoblot signals (Flag/actin) relative to G1/S phase is shown below each lane. (B) HCT116 cells transfected with Flag-tagged ZFP36L2 were treated with 20 µM CDDP for the indicated times after CDDP addition and immunoblotted with an anti-Flag antibody at the respective time points. All experiments shown in this figure were replicated independently at least three times with reproducible results. Fold increase of ZFP36L2 immunoblot signals relative to time zero is indicated under the figure.

We noticed that ZFP36L2 protein levels were moderately but significantly up-regulated in double thymidine treatment-induced S-phase-arrested cells (Figs 1A,B and 6A) compared with the case in G1/S- or G2-phase-arrested cells. Therefore, we examined the possible relationship between DNA replication defects and the amount of ZFP36L2 protein. Cisplatin (cis-Diaminodichloroplatinum, CDDP) has a high affinity for DNA, leading to the formation of bivalent inter- and intra-strand DNA adducts (Basu and Krishnamurthy, 2010; Sears et al., 2016). These crosslinks distort the double helical configuration and perturb DNA replication, thereby stimulating the DNA damage response pathway (Ciccia and Elledge, 2010). When we treated HCT116 cells with 20 µM CDDP, we found that CDDP greatly stimulated the accumulation of ZFP36L2 protein within a short period (Fig. 6B). These observations suggest that ZFP36L2 is a rapid response protein for DNA lesion or DNA replication stresses induced by DNA crosslinking or nucleotide pool depletion.

**Fig. 7.**
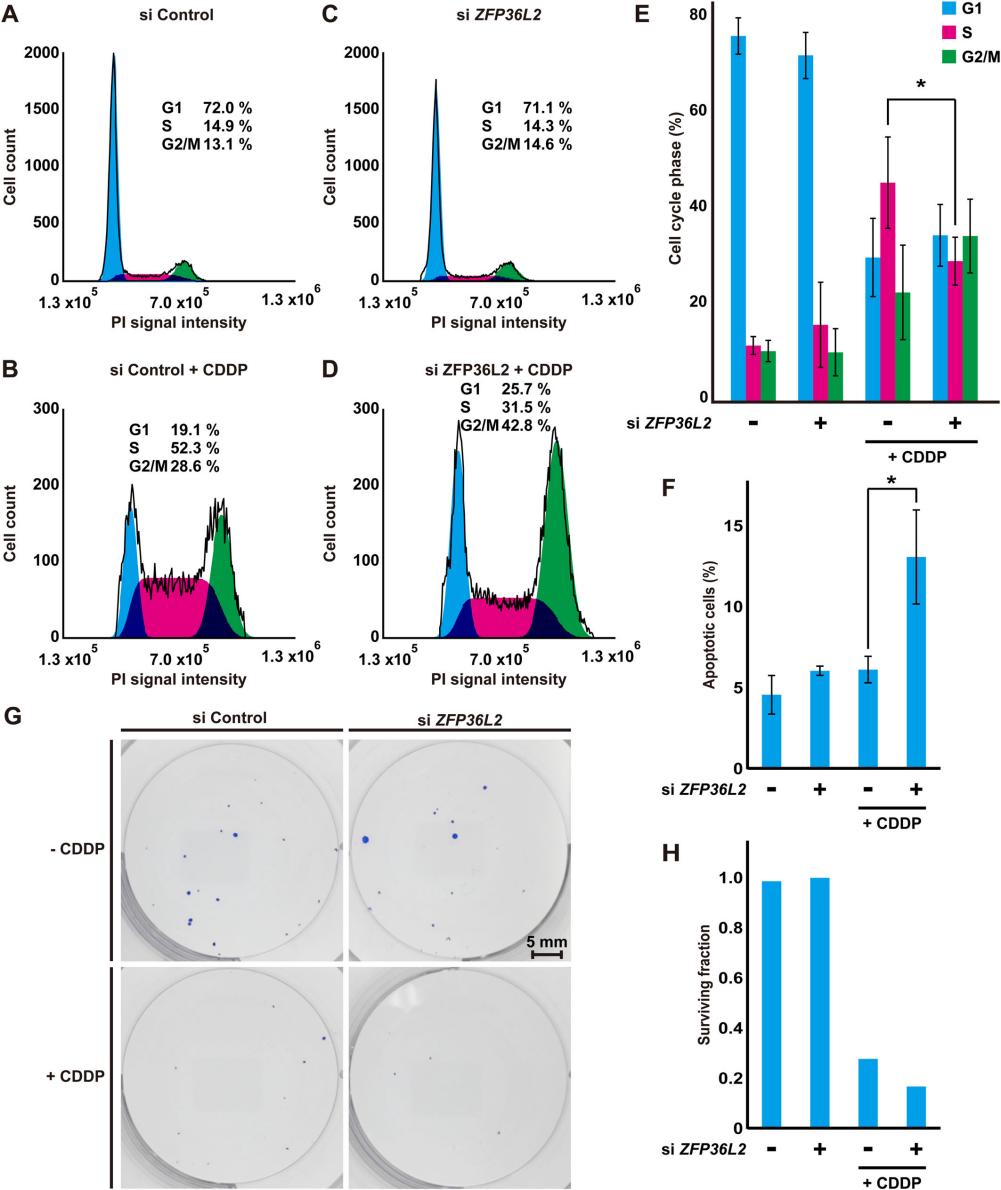
**Depletion of endogenous ZFP36L2 reduces DNA lesion-induced S-phase arrest.** (A-D) Flow cytometric analyses of cell cycle distribution in DNA-damaged HCT116 cells by CDDP treatment. After 24 h of transfection with control siRNA (A,B) or *ZFP36L2* siRNA (C,D), the cells were treated with (B,D) or without (A,C) 20 µM CDDP for 48 h. The cells were harvested and their cell cycle profiles were analyzed using a flow cytometer with propidium iodide (PI) staining. Efficacy of *ZFP36L2* knockdown was verified by anti-Flag-ZFP36L2 immunoblot analysis (see also Fig. S3C). (E) The cell cycle distribution data were quantified and represented as mean±s.d. calculated from three independent biological replicates. *P<0.05 compared with control siRNA cells. (F) Depletion of endogenous ZFP36L2 enhances cell death in the presence of DNA lesions. Apoptotic cells induced by CDDP treatment were estimated by quantifying the activation status of the apoptosis executive protein caspase-3. HCT116 cells were transfected with *ZFP36L2* or control siRNA, treated with or without 20 µM CDDP for 48 h and assessed for active caspase-3 immunostaining by flow cytometry with a PE anti-active caspase-3 antibody. Data represent mean±s.d. calculated from three independent biological replicates. *P<0.05 compared with control siRNA cells. All experiments shown in this figure were replicated at least three times. (G) Image showing colonies produced after incubating HCT116 cells for 7 days following the plating of 200 cells (CDDP+) or 100 cells (CDDP−). Cells were treated with or without *ZFP36L2* siRNA treatment as indicated. (H) Average colony count for six independent wells was used to calculate plating efficiency and the surviving fraction (Rafehi et al., 2011).

Since the suppression of G1/S-phase cyclins is necessary for DNA lesion-induced cell cycle arrest (Sancar et al., 2004), and since the accumulation of ZFP36L2 protein is stimulated during double thymidine or CDDP treatment-induced DNA replication stress (Fig. 6), we speculated that up-regulated ZFP36L2 protein might be critical for checkpoint execution in DNA damage responses, and we investigated this possibility. We treated HCT116 cells with 20 µM CDDP as a replication stress-inducing agent and examined the possible relationship between DNA damage and the function of endogenous ZFP36L2 protein. As shown in Fig. 7 as a representative, CDDP treatment increased the number of S-phase cells (from 14.9% in control cells to 52.3% in CDDP cells, Fig. 7A,B, siControl), in accordance with the idea that DNA lesion stress induces prominent S-phase arrest. Notably, a lack of endogenous ZFP36L2 led to a significant decrease in the S-phase population of HCT116 cells at 48 h relative to the control siRNA-treated cells upon CDDP-induced stress (from 52.3% S-phase-arrested cells in the control knockdown condition to 31.5% in *ZFP36L2* knockdown cells) (Fig. 7B,D) with an increase in the G2/M population (from 28.6% in control cells to 42.8% G2/M-phase cells in *ZFP36L2* knockdown cells, Fig. 7B,D), suggesting that ZFP36L2-suppressed cells passed to the G2/M phase beyond DNA lesion-induced S-phase arrest. In contrast, ZFP36L2 depletion did not affect S phase cell population until 24 h after CDDP treatment (Fig. S6). In addition, *ZFP36L2* knockdown alone (without CDDP) did not apparently influence the populations of either S-phase or G2/M-phase cells (Fig. 7A,C), in accordance with our previous observation of the lack of any growth defects in *ZFP36L2*-suppressed cells (Fig. 5C). Repeated rounds of these experiments suggested that the knockdown effect of *ZFP36L2* with CDDP treatment on S-phase progression was statistically significant (Fig. 7E).

The failure of cell cycle arrest in the presence of DNA lesions tends to trigger cell death. In agreement with this, we noticed a decrease in cell number when ZFP36L2 expression was suppressed under the DNA lesioning condition (induced by CDDP treatment). To examine whether a lack of endogenous ZFP36L2 has any impact on the proportion of cells undergoing cell death, we compared the activation status of the apoptosis executive protein caspase-3, which is a marker for cells in the early stage of apoptosis. As shown in Fig. 7F, either CDDP treatment or *ZFP36L2* knockdown alone only slightly increased the cleavage of caspase-3. Notably, co-treatment with CDDP and *ZFP36L2* siRNA significantly accelerated caspase-3 cleavage (Fig. 7F) when compared with CDDP treatment without *ZFP36L2* siRNA. In accordance with this result, a clonogenic survival assay (Rafehi et al., 2011) showed significantly reduced colony number under co-treatment of CDDP and *ZFP36L2* siRNA, compared with the case of CDDP treatment alone (Fig. 7G,H), supporting the hypothesis that ZFP36L2 depletion renders cells more sensitive to CDDP. These observations suggest that ZFP36L2-mediated S-phase arrest might be essential for cell survival against CDDP-induced DNA lesions in HCT116 cells.

## DISCUSSION

The levels of mitotic cyclins are known to increase greatly at mid-M phase, while the APC/C-mediated down-regulation of cyclin proteins at late M/G1 phase stimulates the transition to G1/S phase. Thus, cyclins and their regulatory proteins have been found to fluctuate throughout the cell cycle, either in their amounts or in their post-translational modifications (Vodermaier, 2004; Guardavaccaro and Pagano, 2006). Nevertheless, no such kinds of regulation of major vertebrate RNA-binding proteins have been identified with respect to a link with cell cycle control.

In this study, we provide the first evidence that human ZFP36L2 is a novel cell cycle-regulated CCCH protein, the abundance of which varies post-translationally during the respective stages of the cell cycle. It was especially up-regulated in S-phase-arrested cells that had altered interactions with a set of RNA-binding proteins (Figs 1, 4 and 6). Such cell cycle-dependent changes of this RNA-binding protein complex could not be accounted for by the unevenness of protein synthesis efficiency of ZFP36L2 at the respective stages, since both frameshifted (at residue 145, Fig. 1F) and C-terminal truncated (ΔC truncation at residue 260, Fig. 3B,C) mutant ZFP36L2 proteins completely lost their instability and/or cell cycle dependency. It was notable that the amount of endogenous *ZFP36L2* mRNA in HCT116 cells also fluctuated at the respective cell cycle stages, peaking at G1/S phase (Fig. S7). This observation suggests that the total amount of endogenous ZFP36L2 protein might be determined by the equilibrium between its protein synthesis and degradation rates. Further investigation is required of the changes in endogenous ZFP36L2 protein levels during cell cycle, especially in response to the DNA replication stress.

A previous study suggested that ZFP36L1 and ZFP36L2 act redundantly to block lymphocyte proliferation, enforce quiescence, and enable the recombination of immunoglobulin genes during early B-cell development (Galloway et al., 2016). Although we confirmed that ZFP36L2 is dispensable for normal cell growth, we showed that *ZFP36L2* knockdown is sufficient to reduce CDDP-induced S-phase arrest, suggesting that the non-redundant function of ZFP36L2 is essential in the presence of DNA lesions. Recently, the genomic landscape of large cohorts of T-lineage acute lymphoblastic leukemia (T-ALL) was revealed, and a spontaneous frame-shift mutation of *ZFP36L2* (at residue 105) was identified as a putative driver for childhood T-ALL (Liu et al., 2017). Furthermore, it was also reported that deletion of murine *Zfp36l1* and *Zfp36l2* leads to perturbed thymic development and T-cell leukemia (Hodson et al., 2010). During VDJ recombination in double-negative 3 stage thymocytes, it was recently reported that *Zfp36l1/Zfp36l2* double KO mice show defects in the DNA damage response caused by T-cell-specific DNA rearrangement-associated double strand breaks, and differentiation into mature T cells was blocked (Vogel et al., 2016). Vogel et al. (2016) speculated that the function of ZFP36L1/ZFP36L2 is suppressed by an ‘as-yet-unknown’ mechanism relieving the inhibition of target transcripts that promote cell cycle progression during T-cell maturation. In this study, we have provided the first possible answer to their proposed mechanism that might account for the cell cycle- and DNA damage-dependent regulation of ZFP36L2 protein. Indeed, the relatively rapid response of ZFP36L2 protein, known as a component of the mRNA elimination machinery, with CDDP treatment might possess some advantages during an ‘emergency’ over transcription factor-mediated DNA damage responses. The identification of the precise regulatory machinery responsible for G1/S-phase-specific ZFP36L2 modification should be pursued in a subsequent study.

In summary, the findings described in this report represent a novel example for this class of zinc finger proteins, which have never been considered to be regulated in a cell cycle-dependent manner. The elimination of vertebrate ZFP36L2 at the interphase and its accumulation with DNA lesioning may provide a biological switch to regulate its ability to control S-phase progression precisely via modulating the amounts of G1/S-phase cyclin transcripts. The concept of enhanced cell lethality in DNA-damaged cells via ZFP36L2 depletion also provides a promising framework for new therapeutic approaches by developing selective pharmacological targeting of this protein. The critical function of ZFP36L2 in cisplatin treatment in particular might have a huge impact on its comprehensive clinical application, as cisplatin-based chemotherapy is utilized widely as a standard anti-cancer therapy for many common carcinomas, including non-small cell lung cancer, ovarian cancer, esophageal cancer, and cervical cancer. The precise identity and regulatory mechanisms of the ZFP36L2 complex in cell cycle control should be promising prospects in future studies.

## MATERIALS AND METHODS

### Plasmid construction

The cDNAs for *ZFP36L2*, *CCNB1* and *ZYG11B* were amplified by PCR from the transcript of HeLa or HEK293 cells as described methods previously (Minami et al., 2010). The amplicon of *ZFP36L2* was ligated into pCI-neo-3xFlag vector, containing three repeats of Flag tag at its N-terminus (Suzuki and Kawahara, 2016). The amplicon of the others were cloned into the pCI-neo-3xT7 vector, containing three repeats of a T7 tag at its N-terminus. The point mutated or truncated derivatives of ZFP36L2 and cyclin B1 were prepared by inverse PCR. All of the cDNAs were verified by DNA sequencing before transfection experiments.

### Cell culture and drug treatment

HeLa cells (RCB0007, Riken Cell Bank, Tsukuba, Japan) were cultured in Dulbecco's modified Eagle's medium (D-MEM, 043-30085, Wako Pure Chemical Industries, Osaka, Japan) with 10% heat-inactivated calf serum, and HCT116 cells (RCB2979, Riken Cell Bank) were cultured in McCoy's 5A Medium (Gibco, Thermo Fisher Scientific) with heat-inactivated 10% fetal bovine serum under 37°C under 5% CO_2_ atmosphere. Cell line renewals to the Riken original clones were executed every 3 months. For translation inhibition and proteasome inhibition, the cells were treated with 20 µg/ml CHX (033-20993; Wako Pure Chemical Industries) and 10 µM MG-132 (3175-v; Peptide Institute, Osaka, Japan), respectively, as described by Suzuki and Kawahara (2016). In the case of CDDP treatment for DNA lesions, the cells were exposed to 20 µM CDDP (P4394; Sigma-Aldrich) starting at 24 h after cDNA transfection and continuing for the indicated time.

### Transfection and protein expression

For the constitutive expression of Flag-ZFP36L2 protein in HeLa and HCT116 cells, 2.0×10^5^ cells in a six-well dish were transfected with 0.25 µg pCI-neo-based mammalian expression vector encoding *ZFP36L2*. DNA transfection was performed using Hily Max (Dojindo Molecular Technologies, Kumamoto, Japan) or Lipofectamine 2000 (Thermo Fisher Scientific), according to the protocols supplied by the manufacturers. Note that the cell cycle synchronization procedures did not affect the gene expression efficiency of the transfected plasmids (Figs 1C,F and 3B).

### RNA interference

For knockdown analysis of *ZFP36L2*, three independent duplex siRNAs covering the targeted sequences

5′-CCUUCUACGAUGUCGACUUtt-3′ (*ZFP36L2* siRNA#1, SASI_Hs01_00137703),

5'-CCAACCUCAACCUGAACAAtt-3′ (*ZFP36L2* siRNA#2, SASI_Hs01_00137706),

5′-CCUCCUACGGCACCCUUAAtt-3′ (*ZFP36L2* siRNA#3, SASI_Hs01_00137708) were synthesized (Sigma-Aldrich).

The siRNA target sequences specific for *ZYG11B* and *CUL2* mRNAs were also synthesized as below (Sigma-Aldrich):

5′-GUAACAAGUGGAUCCAGCAtt-3′ (*ZYG11B siRNA*, SASI_Mm01_00099364),

5′-CUGAAGAAGCCAUGAUCAAtt-3′ (*CUL2 siRNA*, SASI_Hs_00093148).

MISSION siRNA Universal Negative Control 1 (Sigma-Aldrich) was used as a general negative control in every experiment. Transfections of duplex siRNA with HCT116 cells were performed using Lipofectamine 2000 according to the protocol provided by the manufacturer. The efficacy of each siRNA was verified by western blotting.

### Cell synchronization

The cells were arrested at the early S-phase using a double-thymidine block as described by Whitfield et al. (2002) with slight modifications. Briefly, the cells were treated with 2 mM thymidine (Wako Pure Chemical Industries) for 18 h, released for 9 h, and then retreated with 2 mM thymidine for 17 h. The cells were arrested at the M-phase using a thymidine-nocodazole block (Whitfield et al., 2002; Poxleitner et al., 2008). The cells were treated with 2 mM thymidine for 24 h, released for 3 h, and then retreated with 50 nM nocodazole (Wako Pure Chemical Industries) for 12 h. For G1- or G1/S-phase synchronization, the cells were cultured in serum-free medium for 24 h or treated with 5 µg/ml aphidicolin for 24 h. For G2-phase synchronization, the cells were treated with 10 µM RO-3306 (Sigma-Aldrich) for 20 h. The integrity of cell cycle synchronization at the respective stages was verified by flow cytometric analysis (Fig. S1).

### Immunoprecipitation

Cultured cells were washed with ice-cold phosphate-buffered saline (PBS) and suspended in immunoprecipitation (IP) buffer [20 mM Tris-HCl (pH 7.5), 150 mM NaCl, 5 mM EDTA, 1% Nonidet P-40, 10 mM N-ethylmaleimide and 25 µM MG-132]. The lysate was sonicated, centrifuged at 17,000×***g*** for 10 min at 4°C, and the resulting supernatant was incubated with 4 µl anti-Flag M2 affinity gel (Sigma-Merck-Millipore, Darmstadt, Germany) for 10 min at 4°C. The gel was washed five times with the IP buffer, before the precipitated immunocomplexes were eluted in SDS-PAGE sample buffer and subjected to western blot analysis with the appropriate antibodies (Minami et al., 2010; Suzuki and Kawahara, 2016).

### Western blotting and antibodies

For western blot analyses, whole cell lysates and the immunoprecipitates were subjected to SDS-PAGE and then transferred onto Polyvinylidene Fluoride transfer membrane (Merck-Millipore, Darmstadt, Germany). The membranes were immunoblotted with specific antibodies as indicated and then incubated with horseradish peroxidase-conjugated antibody against mouse or rabbit immunoglobulin (GE Healthcare), followed by detection with Immobilon Western (Merck-Millipore).

The following were used as primary antibodies in this study: anti-Flag M2 monoclonal (F3165, Sigma-Merck-Millipore), anti-T7-tag monoclonal (69522, Merck-Millipore), anti-β-actin (A2066, Sigma-Aldrich), and anti-cyclin B1 (sc-245, Santa Cruz Biotechnology). Secondary antibodies: the horseradish peroxidase (HRP)-conjugated anti-mouse IgG (NA931V, GE Healthcare), HRP-conjugated anti-rabbit IgG (NA934V, GE Healthcare). We tried to assess the reported specificity of anti-ZFP36L2 antibodies, but failed to detect reliable cell endogenous antigen signals with the commercially available antibodies.

### Semi-quantitative RT-PCR

Total RNA was extracted from HeLa and HCT116 cells using a Blood/Cultured Cell Total RNA Extraction Mini Kit (Favorgen Biotech, Ping-Tung, Taiwan). With these total RNAs as templates, a reverse transcription (RT) reaction was performed using the SuperScript^R^ III First-Strand Synthesis System for RT-PCR (Thermo Fisher Scientific) according to the manufacturer's protocol. RT products were subjected to PCR with appropriate primer pairs, and the amplified products were visualized by agarose gel electrophoresis. Normalization of the signal intensities of the amplified cDNAs was determined using the actin (*ACTB*) gene as a standard.

### Quantitative real time PCR

Quantitative real-time PCR was performed using the Applied Biosystems Step One Real-Time PCR System (Applied Biosystems, Thermo Fisher Scientific). Gene expressions were analyzed using TaqMan^®^ Gene Expression Master Mix (Applied Biosystems) with 50 ng of cDNA as templates. *ACTB* (actin transcript) was used as an internal control. Gene-specific probes (Applied Biosystems) were as follows: *CCND1* (Hs00765553_m1), *CCND3* (Hs01017690_g1), *CCNE2* (Hs00180319_m1), *CCNA2* (Hs00996788_m1), *CCNB1* (Hs01030099_m1), *CCNG2* (Hs00171119_m1), *CDK1* (Hs00938777_m1), *CDK2* (Hs01528894_m1), *CDK6* (Hs01026371_m1), *ZFP36L2* (Hs00272828_m1), and *ACTB* (Hs01060665_g1). Relative gene expression was calculated by the relative standard curve or 2^−ΔΔdCt^ relative quantification methods.

### Proteomics analysis

HeLa cells were transfected with Flag-tagged ZFP36L2 expression vectors using Lipofectamine 2000 according to the manufacturer's instruction. At 24 h after transfection, the cell cycles were arrested at G1-, S-, G2 and M-phase, respectively, and cells were clashed with PBS containing 20 mM NEM and lysed with lysis buffer [20 mM 4-(2-hydroxyethyl)-1-piperazineethanesulfonic acid (HEPES), pH 7.5, 150 mM NaCl, 20 mM NEM, 50 mM NaF, 1 mM Na_3_VO_4_, 0.5% digitonin, 1 mM phenylmethylsulfonyl fluoride (PMSF), 5 µg/ml leupeptin, 5 µg/ml aprotinin, and 3 µg/ml pepstatin A] and cleared by centrifugation. The cleared lysate was incubated with anti-Flag M2-agarose beads (Sigma-Aldrich) for 1 h, and the agarose resin was washed three times with wash buffer (10 mM HEPES, pH 7.5, 150 mM NaCl, and 0.1% Triton X-100). The immunoprecipitants were eluted with a Flag peptide (0.5 mg/ml; Sigma-Aldrich) dissolved in wash buffer (Natsume et al., 2002). After concentration by TCA precipitation, the ZFP36L2-associated proteins at various cell cycle stages were dissolved in guanidine hydrochloride and digested with lysyl endopeptidase (Lys-C; Wako Chemicals). All samples were analyzed on a direct nanoflow liquid chromatography system coupled to a time-of-flight mass spectrometer (Adachi et al., 2014). The mass spectrometry and tandem mass spectrometry spectra were obtained in information-dependent acquisition mode and were queried against the NCBI nonredundant database with an in-house Mascot server (version 2.2.1, Matrix Science; Natsume et al., 2002). Immunoprecipitates from cell extracts of cell transfected with Flag-tagged empty vector were used as negative controls. Proteins that were identified at least three independent precipitation trials by two or more peptides with a peptide expectation value of *P*<0.05 were considered as reliable identifications.

### Flow cytometry for cell cycle analysis

For quantification of cell cycle stage distribution, the cells were washed twice with PBS, and harvested by trypsinization from culture plates. The cells were re-suspended in 500 µl PBS, and 1 ml 100% ethanol was subsequently added to the cell suspension. After incubation at 4°C for at least 1 h, the cells were washed three times with PBS. The cells were suspended again in PBS and incubated at 4°C for 30 min. After incubation, the cells were centrifuged and the supernatant was removed. Next, 0.5 ml of 250 U/ml RNase A in PBS were added to cell, and the cells were incubated at room temperature for 20 min. After incubation, 50 µg/ml propidium iodide (PI) was added. The cell cycle profile was analyzed using a flow cytometer (model BD Accuri™ C6, BD Biosciences) by 488 nm excitation.

### Flow cytometry for apoptosis analysis

For PE active caspase-3 apoptosis analysis, the cells were washed twice with cold PBS and re-suspended in Cytofix/Cytoperm solution (BD Biosciences), and then incubated for 20 min on ice. Pelleted cells were washed twice with Perm/Wash buffer (BD Biosciences) and re-suspended in Perm/Wash buffer with a PE rabbit anti-active caspase-3 antibody (BD Biosciences). After 30 min incubation at room temperature, the cells were washed in Perm/Wash buffer, re-suspended in Perm/Wash buffer, and analyzed using a flow cytometer by 488 nm excitation.

### Clonogenic survival assay

HCT116 cells treated with siRNA for 24 h were trypsinized with a 0.25% trypsin/EDTA solution for 10 min. After single cell suspensions were generated, the cells were plated in six-well culture plates. For the control CDDP (−) treatment, 100 cells were plated in each well, while 200 cells were plated in each well for the CDDP (+) samples. The cells were subsequently cultured for 7 days with or without 20 µM CDDP and siRNA treatment. Colonies were fixed with 10% neutral buffered formalin solution for 30 min and stained with 0.01% (w/v) Crystal Violet for 30 min. Colonies containing more than 50 individual cells were counted (Rafehi et al., 2011).

### Statistical analysis

Evaluation of data was performed by Student's *t*-test. All data in the figures are presented as the mean±s.e.m. or s.d. *P*<0.05 is considered statistically significant.

## Supplementary Material

Supplementary information
