# Supplementary material for: ZFP36L2 is a cell cycle-regulated CCCH protein necessary for DNA lesion-induced S-phase arrest
Source: Biol Open. 2018 Feb 15;7(3):bio031575. doi: 10.1242/bio.031575 (PMC5898266; doi:10.1242/bio.031575)
Supplement: Supplementary information [file biolopen-7-031575-s1.pdf]

Supplementary Figures

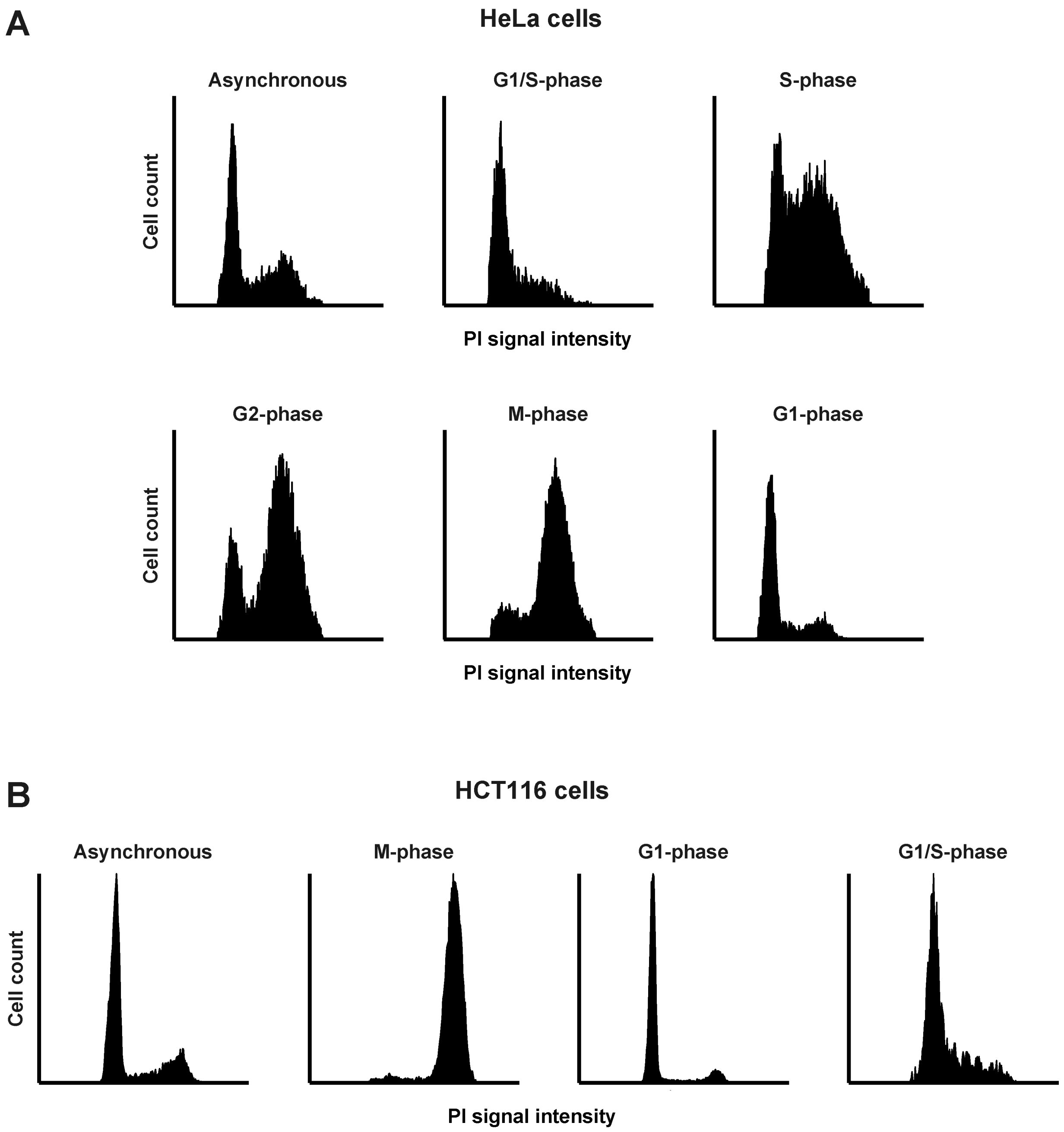

Fig. S1. Related to Figs. 1-4, 6, S2, and S4 and Table S1. Verification of cell cycle synchronization.

HeLa cells (**A**) and HCT116 cells (**B**) were synchronized in G1, G1/S, early S, G2, and M phases, and the efficiency and integrity of cell cycle synchronization at the respective stages was verified by flow cytometric analysis with propidium iodide staining. FACS plots for asynchronous HeLa and HCT116 cells are also given as a baseline for these experiments.

Biology Open • Supplementary information

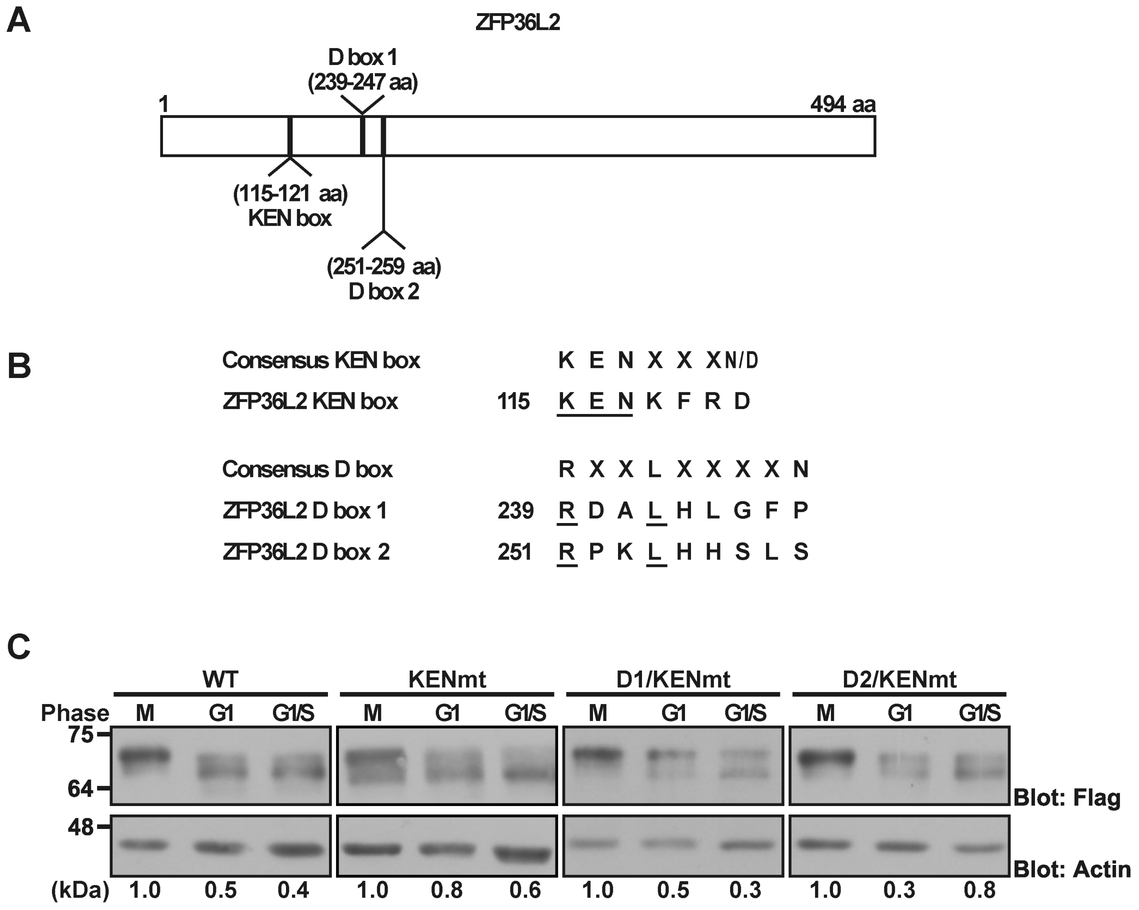

Fig. S2. Related to Fig. 1 and 2; Disruption of either KEN-box-like or D-box-like sequences has only a small effect on the down-regulation of ZFP36L2 protein in the G1-phase.

(A) Schematic diagram and **(B)** amino acid sequences of KEN-box-like and D-box-like motifs in ZFP36L2 protein. Numbers denote the corresponding amino acid numbers of human ZFP36L2. **(C)** Flag-ZFP36L2 and its mutated derivatives were transfected into HeLa cells and synchronized at each cell cycle stage as in Fig. 1A. Levels of ZFP36L2 protein were assessed by western blot analysis with an anti-Flag antibody. Actin was used as a loading control. ZFP36L2 constructs used were mutated in the KEN box (KENmt), D-box 1 and KEN-box (DK1/KENmt), and D-box 2 and KEN-box (DK2/KENmt). Densitometry quantification of Flag-ZFP36L2 immunoblot signals relative to M phase is shown below each lane. Note that some cell cycle-dependent mobility shifts of ZFP36L2 proteins were obvious. The identity of probable post-transcriptional modification(s) of these proteins remains to be addressed.

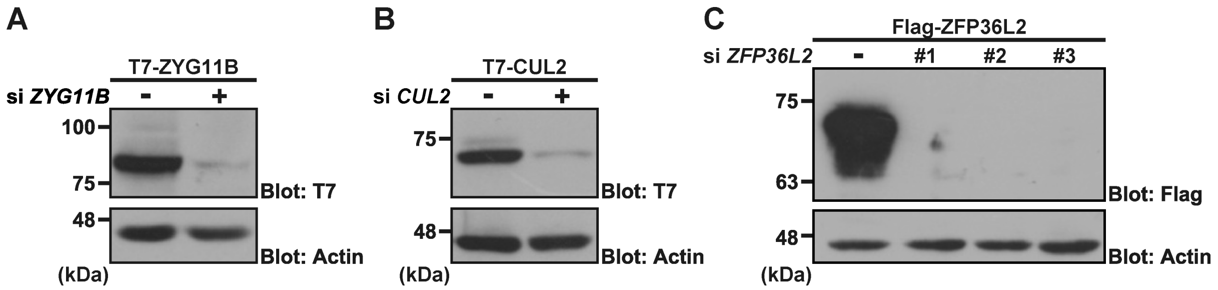

Fig. S3. Related to Figs 2, 5 and 7; Knockdown efficacy of siRNA targeting *ZYG11B*, *CUL2*, and *ZFP36L2*.

Western blots of ectopically expressed T7-ZYG11B, T7-CUL2, and Flag-ZFP36L2 proteins demonstrating the effectiveness of siRNA knockdown of *ZYG11B* (A), *CUL2* (B), and *ZFP36L2* (C, with 3 independent double stranded RNAs), respectively, in HCT116 cells. Actin was used as an internal loading control.

Biology Open • Supplementary information

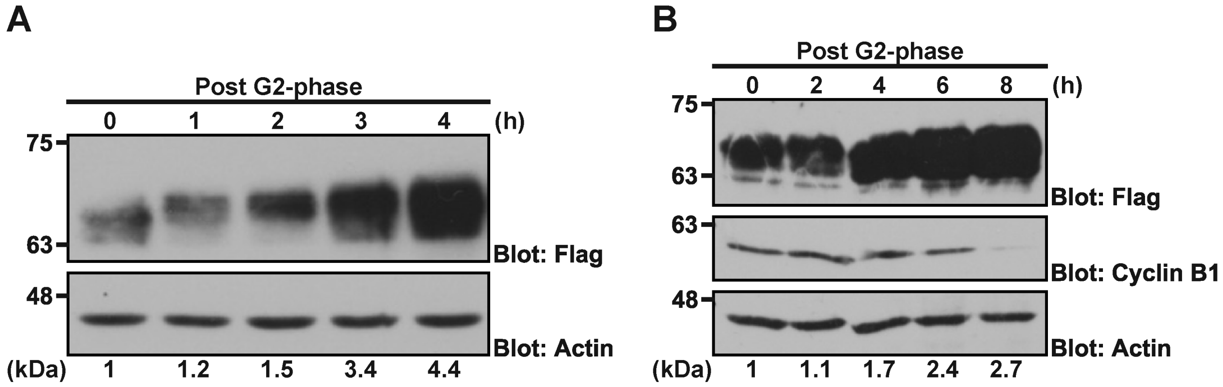

Fig. S4. Related to Fig. 1; ZFP36L2 accumulates in M-phase cells independently to spindle assembly checkpoints.

(A) HeLa cells transfected with Flag-ZFP36L2 were arrested in G2 phase by 10 μM RO-3306, then synchronously released by washing with normal medium. The cells were harvested at the indicated time points after release and blotted with an anti-Flag antibody to quantify the change in Flag-ZFP36L2 during synchronized cell cycle progression. The time when RO-3306-containing medium was replaced by normal medium was defined as time zero. **(B)** Similar experiment as in (A) but with a prolonged chase period. Result of the cyclin B1 immunoblot is also shown. Note that disappearance of cyclin B1 at 8 h does not corresponding exactly to elimination of ZFP36L2. Fold increase of Flag-ZFP36L2 immunoblot signals relative to time zero is indicated under the figure.

Biology Open • Supplementary information

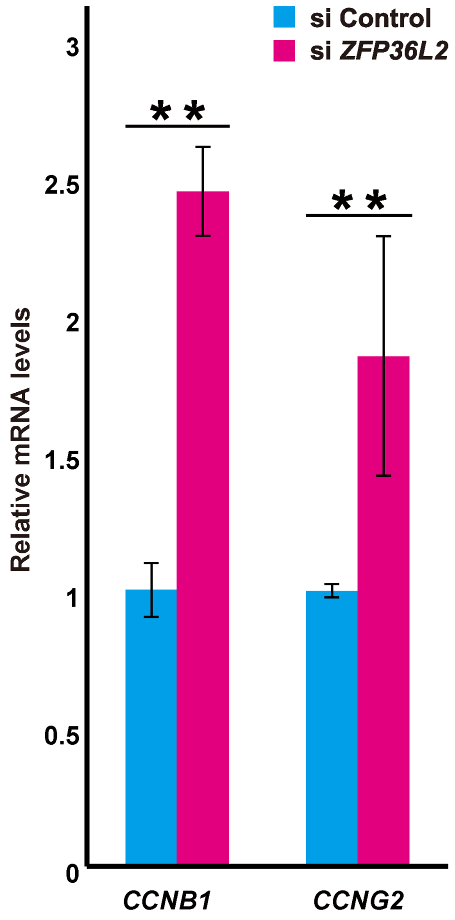

Fig. S5. Related to Fig. 5B; Results of quantitative real-time RT-PCR analyses for *CCNB1* and *CCNG2* in *ZFP36L2* knockdown HCT116 cells.

Knockdown of endogenous *ZFP36L2* stimulates the expression of cyclin B1 (*CCNB1*) and cyclin G2 (*CCNG2*) transcripts. Data represent mean ± S.D. calculated from 3 independent biological replicates. **P < 0.01 compared with control siRNA cells.

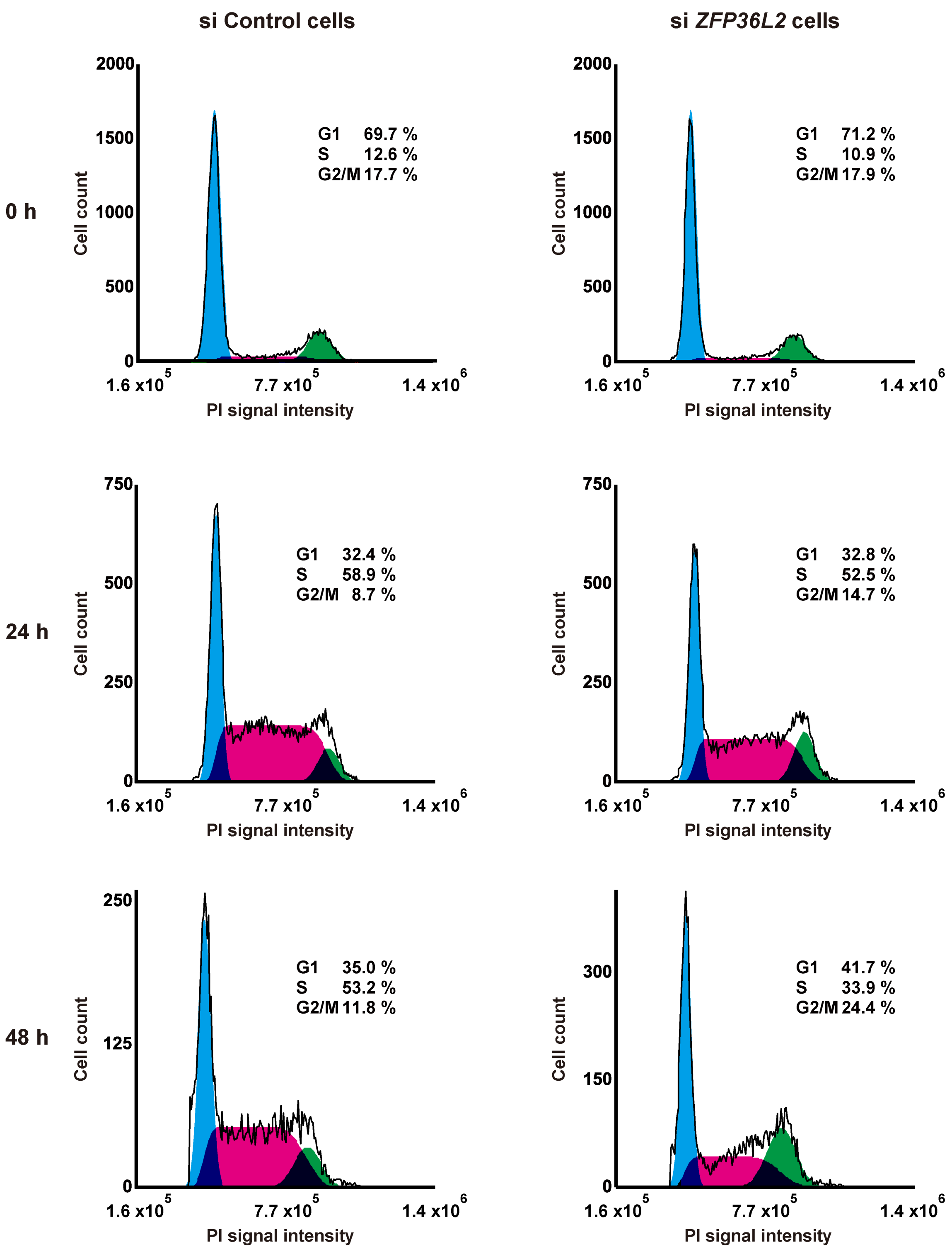

Fig. S6. Related to Fig. 7A-E; Flow cytometric analyses of cell cycle distribution in DNA-damaged HCT116 cells.

After 24 h of transfection with control siRNA (*si Control*) or *ZFP36L2* siRNA (*si ZFP36L2*), the cells were treated with 20 μM CDDP for 0, 24, and 48 h. The cells were harvested and their cell cycle profiles were analyzed using a flow cytometer with propidium iodide (PI) staining.

Biology Open • Supplementary information

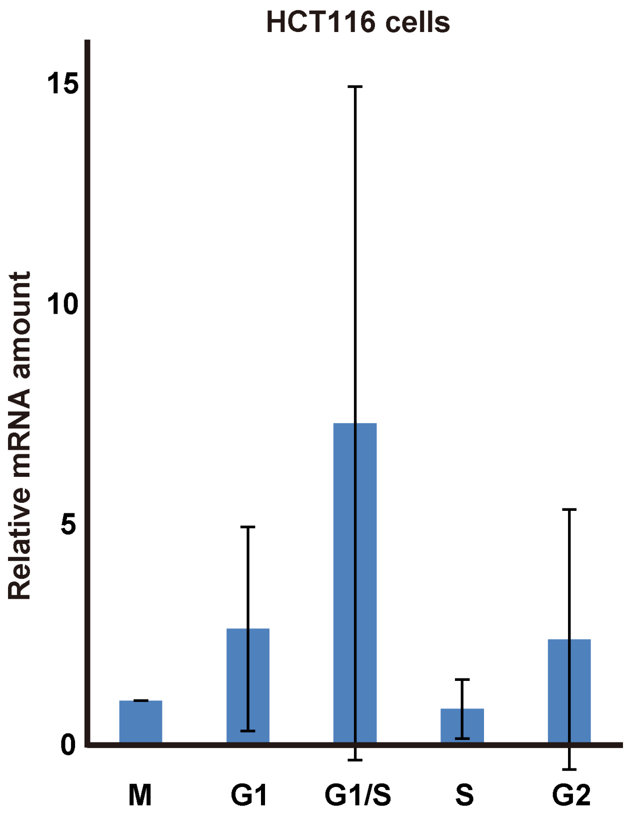

Fig. S7. Levels of endogenous *ZFP36L2* transcripts at the various cell cycle stages.

HCT116 cells were synchronized at G1/S phase, early S phase, G2 phase, M phase, and G1 phase. Amounts of endogenous *ZFP36L2* transcripts in each cell cycle stage were quantified by $2^{-\Delta\Delta dCt}$ relative quantification methods.

Biology Open • Supplementary information

Table S1.

Related to Fig. 4; A full list of ZFP36L2-associated proteins at various cell cycle stages identified by mass spectrometry

N-terminal Flag-tagged ZFP36L2 was expressed in HeLa cells, and Flag-precipitates were subjected to mass spectrometry analysis. Immunoprecipitates from extracts of cells transfected with a Flag-tagged empty vector were used as negative controls. Proteins that were identified in at least 3 independent precipitation trials by 2 or more peptides with a peptide expectation value of $P < 0.05$ were considered reliable identifications.

Biology Open • Supplementary information

Supplemental Table S1

Prey_proteins:		G1S	G1S	S	S	G2	G2	M	M	Mock	Mock
ACTA1|2|B|BL2|C1|G1|G2|POTEE|F|KP	NOT mergeable descriptions	0	1	0	0	0	1	1	0	0	0
ACTA1|A2|B|C1|G1|G2|POTEE|F|I|J	NOT mergeable descriptions	1	1	1	1	1	1	1	1	1	0
ACTB|ACTBL2|ACTG1	actin, cytoplasmic 1 or beta-actin-like protein 2 or actin	0	0	1	1	0	0	1	1	0	0
ACTB|ACTG1	actin, cytoplasmic; 1 or 2	2	2	2	1	1	1	2	1	1	1
ACTB|ACTG1|POTEM	NOT mergeable descriptions	0	0	0	0	0	1	0	0	0	0
AKR1C2	aldo-keto reductase family 1 member C2; isoform 1	0	0	0	0	0	0	1	0	0	0
ARSK	arylsulfatase K; isoform X1	0	0	0	0	0	0	1	1	0	0
ATAD3A|ATAD3C	ATPase family AAA domain-containing protein; 3A or 3C	0	0	0	1	0	0	0	0	0	0
ATP2C1	calcium-transporting ATPase type 2C member 1	0	0	0	0	1	0	0	0	0	0
ATP5B	ATP synthase subunit beta, mitochondrial	0	0	2	1	0	0	0	0	1	1
BANF1	barrier-to-autointegration factor; isoform X1	1	2	1	1	0	0	0	0	1	1
BASP1	brain acid soluble protein 1	0	0	1	1	1	2	1	1	1	0
BRD3	bromodomain-containing protein 3; isoform X2	0	0	0	0	1	1	0	0	0	0
BRSK1	serine/threonine-protein kinase BRSK1; isoform X2	0	1	0	0	0	0	0	0	0	0
C1QBP	complement component 1 Q subcomponent-binding protein,	0	1	1	2	1	0	1	1	1	1
C1R	complement C1r subcomponent; isoform unknown	0	0	0	0	0	0	1	2	0	0
C3	complement C3	1	3	1	2	2	2	1	1	0	3
CALM1|CALM2|CALM3	calmodulin	0	0	0	0	0	1	0	0	0	0
CALM1|CALM2|CALM3|CALML3	calmodulin	1	1	1	1	1	0	1	1	1	1
CALM2|CALM3	calmodulin	0	1	1	0	0	0	0	0	0	0
CAMK2A	calcium/calmodulin-dependent protein kinase type II subunit alpha	1	1	1	0	0	0	0	0	0	0
CAPRIN1	caprin-1	1	0	1	1	0	0	1	1	0	0
CARS2	probable cysteine--tRNA ligase, mitochondrial; isoform X1	0	0	0	0	0	0	1	0	0	0
CCAR2	cell cycle and apoptosis regulator protein 2; isoform unknown	0	0	1	1	0	0	0	0	0	0
CCT6A	T-complex protein 1 subunit zeta; isoform a	0	0	0	1	0	0	0	0	0	0
CENPQ	centromere protein Q; isoform X2	1	0	0	0	0	0	0	0	0	0
CEP170|CEP170P1	centrosomal protein; of 170 kDa	0	0	0	1	0	0	0	0	0	0
CFL1	cofilin-1	0	0	1	1	0	0	0	0	0	0
CHM	rab proteins geranylgeranyltransferase component A 1; isoform X1	0	0	0	0	0	0	0	1	0	0
CMYA5	cardiomyopathy-associated protein 5	1	0	0	0	0	0	0	0	0	0
CSN2	beta-casein precursor ¥[Bos taurus¥]	1	0	0	0	0	0	0	0	0	0
CSNK1A1|HLCDGP1	NOT mergeable descriptions	0	0	1	1	1	0	0	0	0	0
CTNNA1	catenin alpha-1	1	0	0	0	0	0	0	0	0	0
DDX17|DDX5	probable ATP-dependent RNA helicase; DDX17	0	0	0	0	0	0	0	1	0	0
DDX20	probable ATP-dependent RNA helicase DDX20	1	0	2	0	0	0	0	0	0	3
DDX5	probable ATP-dependent RNA helicase DDX5; isoform X3	0	0	0	0	0	0	1	1	0	0
DGCR2	integral membrane protein DGCR2/IDD	1	0	0	0	0	0	0	0	0	0
DHX40	probable ATP-dependent RNA helicase DHX40; isoform X3	0	0	1	0	0	0	0	0	0	0
DLD	dihydrolipoyl dehydrogenase, mitochondrial; isoform unknown	8	8	9	8	4	5	6	6	8	6
DLST	dihydrolipoyllysine-residue succinyltransferase component	4	5	5	4	4	4	3	3	4	4
DYNLRB1|DYNLRB2	dynein light chain roadblock-type; 1	0	0	0	1	0	0	0	0	0	0
EDARADD|ENO1	NOT mergeable descriptions	0	0	1	1	1	1	1	1	1	1
EEF1A1|EEF1A1P5	elongation factor 1-alpha 1	0	0	0	1	1	0	1	0	0	0
EEF1A1|EEF1A1P5|EEF1A2	elongation factor 1-alpha 1	4	2	5	3	3	3	2	1	3	3
EEF1D|EEF1DP1	elongation factor 1-delta	0	0	0	1	0	0	0	0	0	0
EEF2	elongation factor 2	0	0	1	0	0	0	0	1	0	1
EIF3F	eukaryotic translation initiation factor 3 subunit F	0	0	0	0	0	0	0	1	0	0
EIF3F|EIF3FP3	eukaryotic translation initiation factor 3 subunit F; or pseudogene 3	1	0	2	1	1	0	0	0	0	0
EIF3G	eukaryotic translation initiation factor 3 subunit G	0	1	0	1	0	0	0	0	0	0
EIF3H	eukaryotic translation initiation factor 3 subunit H	0	0	0	0	0	0	1	0	0	0
EIF3M	eukaryotic translation initiation factor 3 subunit M; isoform X1	0	0	1	0	0	0	1	1	0	0
EIF4A1|EIF4A2	eukaryotic initiation factor; 4A-I	0	0	1	0	1	0	1	0	0	1
EIF4B	eukaryotic translation initiation factor 4B; isoform 2	12	9	10	8	9	9	6	5	8	10
EIF5A|EIF5A2|EIF5AL1	eukaryotic translation initiation factor; 5A-1	0	0	1	1	1	1	1	1	1	1
EIF5A|EIF5AL1	eukaryotic translation initiation factor; 5A-1	0	0	0	1	0	0	0	0	0	0
ENO1	alpha-enolase; c-myc promoter-binding protein-1; isoform X1	2	1	4	3	1	1	1	0	1	1
ENO1|ENO1B	alpha-enolase; c-myc promoter-binding protein-1	0	1	1	1	1	1	1	1	1	1
ENO1|ENO1B|ENO3|MPB1	NOT mergeable descriptions	0	0	1	1	0	1	1	1	1	1
ENO1|ENO1B|MPB1	NOT mergeable descriptions	1	1	1	1	1	1	1	1	1	1
ENO1|ENO2|ENO3	alpha-enolase; c-myc promoter-binding protein-1	1	1	2	2	0	0	1	2	2	1
ENO1|MPB1	promoter binding protein family	0	0	0	0	0	0	0	1	0	0
EZR	ezrin	0	0	0	1	0	1	0	0	0	0
EZR|MSN|RDX	ezrin family	0	1	1	0	0	1	1	1	0	1
FAM160B1	protein FAM160B1	0	1	0	0	1	0	0	0	0	0
FAM57B	protein FAM57B; isoform 2	0	0	0	1	0	0	0	0	0	0

Biology Open • Supplementary information

FBXO11	F-box only protein 11; isoform unknown	0	0	0	0	0	0	1	0	0	0
FLJ10833|SBNO1	FLJ10833 family	0	0	0	0	0	0	1	0	0	0
FLJ25054	hypothetical protein FLJ25054	0	0	0	0	0	0	0	1	0	0
FLJ27099|IGHG1|IGHG2	NOT mergeable descriptions	1	1	0	0	0	0	0	0	0	0
FLJ27099|IGHG2|IGHM	NOT mergeable descriptions	1	1	1	1	1	1	1	1	1	1
FLJ27099|IGHG2	NOT mergeable descriptions	1	1	0	0	0	0	0	0	0	0
FLNA|FLNB|FLNC	filamin-A	0	0	1	1	0	0	0	1	1	1
FLOT1	flotillin-1; isoform X4	0	0	0	0	0	0	1	0	0	0
FYTTD1	UAP56-interacting factor; isoform 1	0	0	1	1	0	0	0	0	0	0
G3BP1	ras GTPase-activating protein-binding protein 1; isoform X1	0	0	0	0	0	0	0	1	0	0
GAPDH	glyceraldehyde-3-phosphate dehydrogenase; isoform unknown	6	3	7	6	4	3	6	5	5	5
GAS1	growth arrest-specific protein 1	1	0	0	0	0	0	0	0	0	0
GFAP|KRT1|4|7|8|80|8P3|8P44|NCKAP1	NOT mergeable descriptions	0	0	0	0	1	1	0	0	0	0
GPI	glucose-6-phosphate isomerase; isoform X3	0	0	0	1	0	0	0	0	0	0
HADHA	trifunctional enzyme subunit alpha, mitochondrial	0	0	0	0	0	0	1	0	0	0
HBA1|HBA2	hemoglobin subunit alpha;	1	1	1	1	0	1	1	0	1	0
HBB|HBD|HBE1|HBG1|HBG2	hemoglobin subunit; beta or delta or epsilon or gamma-1 or gamma-2	0	0	0	0	0	0	0	1	0	0
HEXDC	hexosaminidase D; isoform X3	0	1	0	0	0	0	0	0	0	0
HNRNPA1|HNRNPA1L2	heterogeneous nuclear ribonucleoprotein; A1	0	0	0	0	0	0	1	0	0	0
HNRNPA1|HNRNPA1L2|HNRNPA1P8	heterogeneous nuclear ribonucleoprotein; A1	0	0	0	0	0	0	1	1	0	0
HNRNPA2B1	heterogeneous nuclear ribonucleoproteins A2/B1; isoform X2	0	0	0	2	1	2	7	7	0	0
HNRNPAB	heterogeneous nuclear ribonucleoprotein A/B; isoform a	0	0	0	0	0	0	2	2	0	0
HNRNPC|HNRNPCL1|CL2|CL3|CP2	heterogeneous nuclear; ribonucleoproteins C1/C2	0	0	0	0	0	0	0	1	0	0
HNRNPD|HNRNPDL	heterogeneous nuclear ribonucleoprotein; D0	0	0	0	0	0	0	1	0	0	0
HNRNPDL	heterogeneous nuclear ribonucleoprotein D-like; isoform a	0	0	0	0	0	0	1	0	0	0
HNRNPH1|HNRNPH2	heterogeneous nuclear ribonucleoprotein; H	0	0	0	0	0	0	1	1	0	0
HNRNPK	heterogeneous nuclear ribonucleoprotein K; isoform a	5	4	8	10	6	5	6	7	0	0
HNRNPL	heterogeneous nuclear ribonucleoprotein L; isoform X5	1	2	1	1	1	2	3	4	0	0
HNRNPL|HNRNPLL	heterogeneous nuclear ribonucleoprotein; L	0	0	0	1	0	0	1	0	0	0
HNRNPM	heterogeneous nuclear ribonucleoprotein M; isoform X4	0	0	0	1	0	0	0	0	0	0
HNRNPR|SYNCRIP	heterogeneous nuclear ribonucleoprotein family	2	1	2	2	2	2	1	2	0	0
HNRNPU	heterogeneous nuclear ribonucleoprotein U; isoform a	0	0	0	0	0	0	1	1	0	0
HSP90AA1	heat shock protein HSP 90-alpha; isoform X1	1	1	0	0	0	0	0	0	0	0
HSP90AA1|AA2P|AB1|AB2P	heat shock protein; HSP 90-alpha (isoform unknown)	0	1	1	1	2	1	1	1	1	2
HSP90AA1|AA5P|AB1|AB3P|AB4P	heat shock protein; HSP 90-alpha	0	1	1	0	0	1	0	1	0	1
HSP90AA1|AB1|AB3P	heat shock protein; HSP 90-beta or HSP 90-alpha (isoform unknown)	0	0	1	1	0	0	0	0	0	0
HSP90AB1|AB2P	heat shock protein; HSP 90-beta	1	1	1	1	0	0	0	0	0	0
HSP90AB1|AB3P	heat shock protein; HSP 90-beta	0	0	2	2	0	0	0	0	0	1
HSP90AB1|AB6P	heat shock protein; HSP 90-beta	0	0	0	1	0	0	0	0	0	0
HSP90AB1|B1	heat shock protein HSP 90-beta	0	0	0	0	1	0	0	1	0	0
HSPA1A|1B	heat shock 70 kDa protein; 1A or 1B	0	1	0	0	0	0	0	0	0	0
HSPA1A|1B|1L|2|6|7|8	heat; shock 70 kDa protein 1A or 1B	1	0	1	1	0	0	1	0	0	0
HSPA1A|1B|6|7	heat shock; 70 kDa protein 1A or 70 kDa protein 1B	0	0	0	0	1	0	1	0	0	0
HSPA1L|HSPA2|5|8	heat shock 70 kDa protein 1-like	1	1	1	1	0	1	1	0	1	0
HSPA2|HSPA8	heat; shock-related 70 kDa protein 2 or shock cognate 71 kDa protein	1	2	1	1	1	2	2	2	1	1
HSPA8	heat shock cognate 71 kDa protein; isoform 2	1	1	3	2	3	1	2	2	2	1
HSPA8|HSPA8P8	heat shock; cognate 71 kDa protein	0	2	2	2	2	2	1	2	1	0
HSPA9	stress-70 protein, mitochondrial	0	0	0	0	1	0	1	0	1	0
HSPD1	60 kDa heat shock protein, mitochondrial	2	1	5	5	2	3	4	3	4	4
HSPE1	10 kDa heat shock protein, mitochondrial	0	0	1	0	0	0	0	0	0	0
IGHG1	immunoglobulin heavy constant gamma 1 (G1m marker)	0	1	3	1	0	0	0	0	0	0
IGK|IGKC|IGKV1-5|PDPK1	NOT mergeable descriptions	1	1	1	0	0	1	1	1	1	1
IGKV2-26|IGKV2-29|IGKV2D-26	immunoglobulin kappa variable; 2-26 (pseudogene)	1	1	1	1	1	1	1	1	1	1
IRF6	interferon regulatory factor 6	0	0	1	0	0	0	0	0	0	0
ITGB3	integrin beta-3	0	0	0	0	1	0	0	0	0	0
IVNS1ABP	influenza virus NS1A-binding protein; isoform X1	0	0	5	6	0	0	0	1	1	1
JCHAIN	immunoglobulin J chain	0	0	1	0	0	0	1	0	0	0
KHDRBS1	KH domain-containing, RNA-binding, signal transduction-associated	0	0	1	1	0	0	1	0	0	0
LCTL	lactase-like protein; isoform 1 precursor	0	1	0	0	0	0	0	0	0	0
LDHA	L-lactate dehydrogenase A chain; isoform 1	3	1	3	4	4	2	2	4	3	2
LDHA|LDHAL6B	L-lactate dehydrogenase; A chain	0	1	1	1	1	1	0	0	1	1
LDHB	L-lactate dehydrogenase B chain; isoform LDHB or KLRF1-s	1	0	4	3	0	2	0	2	1	1
LOC100287166	uncharacterized LOC100287166	0	0	0	0	0	1	0	0	0	0
LOC101928892	nascent polypeptide-associated complex subunit alpha,; isoform X2	1	0	0	0	0	0	0	0	0	0
LOC102724426	Sjoegren syndrome nuclear autoantigen 1 homolog	0	0	0	0	1	0	0	0	0	0
LOC151174	uncharacterized LOC151174	0	0	0	0	0	1	0	0	0	0
LOC388817|PPIA	isomerase family	4	2	5	7	1	3	3	2	4	3
LOC388817|PPIA|PPIAL4C	isomerase family	0	0	0	1	0	0	0	0	0	0
LOC51781|LOC51782|OGFR	opioid growth factor receptor family	0	0	0	1	0	0	0	0	0	0

Biology Open • Supplementary information

LOC643576|PGAM1|1P4|2	phosphoglycerate mutase family	0	0	0	1	0	0	0	0	0	0
LRPPRC	leucine-rich PPR motif-containing protein, mitochondrial; isoform X3	20	30	26	27	18	20	12	12	0	0
LRRC9	leucine rich repeat containing 9	0	0	0	0	1	0	0	0	0	0
LTF	lactotransferrin	1	1	0	1	0	1	1	0	1	1
MAB21L1	protein mab-21-like 1	0	0	0	0	0	1	0	0	0	0
MDH2	malate dehydrogenase, mitochondrial; isoform 1 precursor	1	0	1	1	0	0	1	0	0	0
MED16	mediator of RNA polymerase II transcription subunit 16	0	0	0	1	0	0	0	0	0	0
MGA	MAX gene-associated protein; isoform X11	1	0	0	0	0	0	0	0	0	0
MGC15885	uncharacterized protein MGC15885	0	1	0	0	0	0	0	0	0	0
MIF	macrophage migration inhibitory factor	0	0	0	0	0	0	0	1	0	0
MRPL12	39S ribosomal protein L12, mitochondrial	1	1	1	1	1	0	1	1	0	1
MRPS36	28S ribosomal protein S36, mitochondrial	2	0	1	2	0	0	0	0	1	1
MYBBP1A	myb-binding protein 1A; isoform X1	0	0	0	0	0	0	1	0	0	0
MYCBP	C-Myc-binding protein	0	0	2	1	1	0	1	0	2	3
MYL6	myosin light polypeptide 6; isoform 2	1	0	1	1	0	0	1	0	1	1
MYL6|MYL6B	myosin light; polypeptide 6	0	0	0	1	0	0	0	1	0	0
NCL	nucleolin	2	1	2	1	1	2	4	5	2	1
NEDD4	E3 ubiquitin-protein ligase NEDD4; isoform 2	0	0	0	0	0	0	2	2	0	0
NEDD4|NEDD4L	E3 ubiquitin-protein ligase; NEDD4	0	0	0	0	0	0	2	2	0	0
NME1	nucleoside diphosphate kinase A; isoform a	0	0	1	1	0	0	0	0	0	0
NME1-NME2|NME2	NME1-NME2 protein or nucleoside diphosphate kinase B	0	0	1	0	0	0	0	0	0	0
NME1|NME1-NME2|NME2	nucleoside diphosphate kinase A	0	1	0	1	0	0	1	0	0	0
NONO	non-POU domain-containing octamer-binding protein; isoform 2	2	1	1	1	1	1	1	2	0	0
NPM1	nucleophosmin; isoform X1	0	0	1	0	0	0	0	0	0	0
OGDH	2-oxoglutarate dehydrogenase, mitochondrial; isoform X2	13	11	9	8	6	3	8	9	8	8
OGDH|OGDHL	2-oxoglutarate; dehydrogenase, mitochondrial	4	3	4	2	3	2	3	2	2	3
OTUD4	OTU domain-containing protein 4; isoform X2	1	1	1	1	1	1	0	0	0	1
OTUD4|OTUD4P1	OTU domain-containing protein 4	0	0	1	0	0	0	0	0	0	0
PABP3|PABPC1|1L|1L2B|3	testis-specific poly(A) -binding protein 3	1	0	0	1	0	0	0	0	0	0
PABPC1	polyadenylate-binding protein 1; isoform X1	1	1	1	1	1	1	1	1	0	0
PABPC1|PABPC1L|PABPC5	polyadenylate-binding protein; 1	1	1	1	1	1	1	1	1	0	0
PABPC1|PABPC4	polyadenylate-binding protein; 1	2	1	2	2	0	1	3	0	0	0
PADI1	protein-arginine deiminase type-1; isoform X1	0	0	1	0	0	0	0	0	0	0
PCBP1|PCBP2	poly(rC) -binding protein; 1 or 2	0	0	0	1	0	0	0	0	0	0
PCBP1|PCBP2|PCBP3	poly(rC) -binding protein; 1 or 2	0	0	2	1	0	0	2	1	0	0
PCDHA4	protocadherin alpha-4; isoform unknown	0	0	0	1	0	0	0	0	0	0
PCID2	PCI domain-containing protein 2	1	0	0	1	0	0	0	0	0	0
PFN1	profilin-1	1	1	3	3	1	0	1	2	2	0
PGD	6-phosphogluconate dehydrogenase, decarboxylating; isoform 1	0	0	0	1	0	0	0	0	0	0
PGK1|PGK2	phosphoglycerate kinase; 1 or 2	0	0	1	0	0	0	0	0	0	0
PLCH2	1-phosphatidylinositol 4,5-bisphosphate phosphodiesterase eta-2	0	0	0	0	1	0	0	0	0	0
PLS3	plastin-3; isoform unknown	0	0	0	0	0	0	1	0	0	0
POLR2G	DNA-directed RNA polymerase II subunit RPB7	0	1	0	0	0	0	0	0	0	0
PPIA	peptidyl-prolyl cis-trans isomerase A; isoform 1	0	0	1	1	1	1	1	1	1	1
PPIB	peptidyl-prolyl cis-trans isomerase B	0	0	0	1	0	0	0	0	0	0
PRDX1	peroxiredoxin-1	1	0	1	2	1	1	2	0	1	2
PRDX1|PRDX2	peroxiredoxin-1 or peroxiredoxin-2	1	0	1	1	1	1	1	1	0	0
PRMT5	protein arginine N-methyltransferase 5; isoform unknown	2	1	0	0	0	0	0	0	0	0
PROCA1	protein PROCA1; isoform X5	1	0	0	0	0	0	0	0	0	0
PRPS1	ribose-phosphate pyrophosphokinase 1; isoform 1	0	1	1	1	0	0	0	1	1	0
PRPS1|PRPS1L1	ribose-phosphate pyrophosphokinase; 1	0	0	1	0	0	0	0	0	0	0
PRPS1|PRPS1L1|PRPS2	ribose-phosphate pyrophosphokinase; 1	1	1	2	1	1	0	2	2	1	2
PRPS1|PRPS2	ribose-phosphate pyrophosphokinase; 1	2	3	3	3	2	1	3	3	2	3
PRPS2	ribose-phosphate pyrophosphokinase 2; isoform 2	0	0	1	1	0	0	1	0	1	0
PRPSAP1	phosphoribosyl pyrophosphate synthase-associated protein 1	0	0	8	8	0	0	4	3	1	3
PRPSAP1|PRPSAP2	phosphoribosyl pyrophosphate synthase-associated protein	0	0	1	1	0	0	0	0	1	1
PRPSAP2	phosphoribosyl pyrophosphate synthase-associated protein 2	1	0	7	9	0	0	2	3	1	2
PRSS1	trypsin-1; isoform X1	1	1	1	1	1	1	1	1	1	1
PRSS3|PRSS4	trypsin-3 or protease, serine, 4 (trypsin 4, brain)	0	0	0	0	0	0	1	0	0	0
PRSS4	protease, serine, 4 (trypsin 4, brain)	1	0	0	0	0	0	0	0	0	0
PTBP1	polypyrimidine tract-binding protein 1; isoform X2	0	0	1	3	1	1	2	0	0	0
PTPRS	receptor-type tyrosine-protein phosphatase S; isoform X17	0	0	0	0	1	0	0	0	0	0
PUS10|RPS12	NOT mergeable descriptions	0	0	0	1	0	0	0	1	0	0
PZP	pregnancy zone protein	1	1	1	1	1	0	1	1	1	1
RAB39A	ras-related protein Rab-39A	0	0	1	0	0	0	1	0	0	0
RBM10	RNA-binding protein 10; isoform X6	0	1	1	3	2	1	3	3	0	0
RBMX|RBMXL2	RNA-binding motif protein; X chromosome	0	0	0	0	0	0	1	0	0	0
RBMY1C	RNA-binding motif protein, Y chromosome, family 1 member C	0	0	0	0	0	0	0	1	0	0
REL	proto-oncogene c-Rel; isoform X2	0	0	0	0	1	0	0	0	0	0

RNF219	RING finger protein 219	1	1	0	1	0	0	0	0	0	0
RPL11	60S ribosomal protein L11; isoform 2	0	0	1	0	0	0	1	1	0	0
RPL12|RPL12P1|RPL12P16	60S ribosomal protein L12	0	0	0	1	0	0	1	0	0	0
RPL15	60S ribosomal protein L15; isoform 1	1	1	1	1	1	1	1	1	0	0
RPL22	60S ribosomal protein L22	2	1	1	1	1	2	2	1	0	0
RPL3|RPL3L	60S ribosomal protein; L3	0	0	0	0	0	1	0	1	1	0
RPL35	60S ribosomal protein L35	0	0	1	0	0	0	0	0	0	0
RPL5|RPLP0	60S; ribosomal protein L5 or acidic ribosomal protein P0	0	0	0	0	1	2	1	0	0	0
RPL7|RPL7P23|RPL7P32|RPL7P9	60S ribosomal protein L7	0	1	0	1	1	1	1	1	0	1
RPLP0|RPLP0P6	60S acidic ribosomal protein P0	1	1	1	1	0	0	2	2	1	1
RPLP1	60S acidic ribosomal protein P1; isoform unknown	0	0	0	1	0	0	0	0	0	0
RPLP2	60S acidic ribosomal protein P2	1	0	0	0	0	0	1	2	0	0
RPS10	40S ribosomal protein S10	0	0	0	1	0	1	1	1	0	0
RPS11	40S ribosomal protein S11	0	0	0	0	0	0	1	0	0	0
RPS13	40S ribosomal protein S13	0	1	0	1	0	0	0	1	1	0
RPS15	40S ribosomal protein S15; isoform 2	1	1	1	1	0	0	1	1	0	0
RPS16	40S ribosomal protein S16; isoform 2	0	1	1	1	1	1	1	1	0	0
RPS18	40S ribosomal protein S18	0	0	0	0	0	0	0	1	0	0
RPS19	40S ribosomal protein S19; isoform 1	0	0	0	0	1	0	1	1	0	0
RPS2|RPS2P40|RPS2P5	40S ribosomal protein S2	0	0	0	1	0	0	1	0	0	0
RPS24	40S ribosomal protein S24; isoform X5	0	0	0	1	0	0	1	1	0	0
RPS27A|UBA52|UBB|UBC	NOT mergeable descriptions	1	1	1	2	2	2	1	0	1	1
RPS3	40S ribosomal protein S3; isoform 1	0	0	1	0	0	0	0	3	0	0
RPS3A	40S ribosomal protein S3a; isoform 1	0	0	0	0	1	0	0	0	0	0
RPS4X	40S ribosomal protein S4, X isoform	1	1	0	2	0	0	1	1	0	0
RPS5	40S ribosomal protein S5	0	0	1	1	1	0	1	0	0	0
RPS7	40S ribosomal protein S7	1	0	0	1	0	0	1	0	0	0
RPSA|RPSAP15|19|47|58	40S ribosomal protein SA	0	0	1	1	0	0	2	0	0	0
RPSA|RPSAP15|47	40S ribosomal protein SA	0	0	1	0	0	0	0	0	0	0
RPSA|RPSAP15|58	40S ribosomal protein SA	1	1	0	1	0	0	0	1	1	0
RPSA|RPSAP47|58	40S ribosomal protein SA	0	0	0	1	0	0	0	1	0	0
RPSA|RPSAP58	40S ribosomal protein SA	0	0	0	0	0	0	1	0	0	0
S100A16	protein S100-A16; isoform X2	1	1	1	0	0	0	0	0	0	0
S100A6	protein S100-A6; isoform X1	2	3	3	3	2	0	1	1	2	1
SETBP1	SET-binding protein; isoform a	0	0	0	0	0	1	0	0	0	0
SFN|YWHAB|E|G|H|Q|Z	14 3 protein family	1	1	1	1	1	1	1	1	1	1
SFN|YWHAB|G|H|Q	14 3 protein family	1	0	0	1	1	1	1	1	0	0
SFPQ	splicing factor, proline- and glutamine-rich; isoform unknown	0	0	1	0	0	0	0	0	0	0
SIK3	serine/threonine-protein kinase SIK3	1	0	0	0	0	0	0	0	0	0
SIX1	homeobox protein SIX1; isoform X1	1	0	0	0	0	0	0	0	0	0
SLC38A10	putative sodium-coupled neutral amino acid transporter 10	0	0	0	0	1	0	0	0	0	0
SLIRP	SRA stem-loop-interacting RNA-binding protein, mitochondrial	0	1	3	3	2	4	0	0	0	0
SMN1|SMN2	survival motor neuron; protein or protein (isoform unknown)	2	1	2	1	0	0	0	0	2	1
SPTBN1	spectrin beta chain, non-erythrocytic 1; isoform X2	0	0	0	0	0	0	0	1	0	0
SPTBN5	spectrin beta chain, non-erythrocytic 5	0	1	0	0	0	0	0	0	0	0
STK38	serine/threonine-protein kinase 38; isoform X2	8	6	6	7	4	2	6	5	5	4
STK38|STK38L	serine/threonine-protein kinase; 38	1	1	2	2	1	2	2	1	2	0
STRAP	serine-threonine kinase receptor-associated protein	0	0	0	1	0	0	0	0	1	0
SUPT16H	FACT complex subunit SPT16; isoform X1	0	0	0	1	0	0	0	0	0	0
SYNCRIP	heterogeneous nuclear ribonucleoprotein Q; isoform X6	2	2	6	4	2	2	2	2	0	0
TAGLN2	transgelin-2; isoform a	0	0	3	4	0	0	1	0	3	2
TENM1	teneurin-1; isoform X2	0	0	0	1	0	0	0	0	0	0
TET1	methylcytosine dioxygenase TET1; isoform X6	0	0	0	0	0	0	1	0	0	0
THRAP3	thyroid hormone receptor-associated protein 3; isoform 1	0	0	0	0	0	0	2	0	0	0
TKT	transketolase; isoform 2	1	1	1	2	1	1	1	2	1	1
TMPO	thymopoietin	1	1	0	0	0	0	0	0	0	0
TOP1MT	DNA topoisomerase I, mitochondrial	1	0	0	0	0	0	0	0	0	0
TPI1	triosephosphate isomerase; isoform 3	0	0	2	1	0	0	0	0	0	0
TPI1|TPI1P1	triosephosphate; isomerase	0	0	1	1	0	0	0	0	0	0
TPPP	tubulin polymerization-promoting protein; isoform X2	0	0	0	1	0	0	0	0	0	0
TPT1P8	tumor protein, translationally-controlled 1 pseudogene 8	1	1	0	1	1	0	1	1	1	1
TRAPPC9	trafficking protein particle complex subunit 9; isoform unknown	0	0	0	0	0	0	0	1	0	0
TRIM21	E3 ubiquitin-protein ligase TRIM21	1	1	2	1	0	0	0	0	0	0
TRIM31	E3 ubiquitin-protein ligase TRIM31; isoform X4	0	1	0	0	0	0	0	0	0	0
TRIM47	tripartite motif-containing protein 47	0	0	1	0	0	0	0	0	0	0
TTK	dual specificity protein kinase TTK or KLRF1-s	0	0	0	0	0	0	0	1	0	0
TUBA1B|1C|4A|4B|8	tubulin alpha-1B chain or tubulin alpha-1C chain	1	1	1	0	0	1	1	0	1	1
TUBB|TUBB2A|2B|3|4A|4B	tubulin; beta chain	0	0	0	1	0	0	0	0	0	0
TUBB|TUBB2A|2B|3|4B|6	tubulin; beta chain	0	1	0	0	0	0	0	0	0	0

Biology Open • Supplementary information

TUBB|TUBB2A|2B|4A|4B	tubulin; beta chain	0	0	0	1	0	0	0	0	0	0
TUBB|TUBB4A	tubulin; beta chain (isoform unknown) or beta-4A chain	0	0	0	1	0	0	0	0	0	0
TXN	thioredoxin; isoform 1	0	0	2	2	0	1	0	0	2	1
UACA	uveal autoantigen with coiled-coil domains and ankyrin repeats	0	1	1	0	0	0	0	0	0	0
UBR3	E3 ubiquitin-protein ligase UBR3; isoform X7	0	1	0	0	0	0	0	0	0	0
UNC45B	protein unc-45 homolog B; isoform 1	0	1	0	0	0	0	0	0	0	0
USP9Y	probable ubiquitin carboxyl-terminal hydrolase FAF-Y; isoform X2	0	0	0	0	0	0	0	1	0	0
YBX1	nuclease-sensitive element-binding protein 1	2	1	0	1	2	1	1	1	0	0
YBX1|YBX2|YBX3	nuclease-sensitive element-binding protein 1 or Y-box-binding protein	0	0	0	0	0	1	0	0	0	0
YWHAB	14-3-3 protein beta/alpha; isoform X1	3	3	6	2	4	3	3	4	0	0
YWHAB|YWHAG	14-3-3 protein; beta/alpha	0	1	2	2	2	1	2	1	0	0
YWHAE	14-3-3 protein epsilon; isoform X1	2	3	6	4	3	5	7	4	1	1
YWHAE|YWHAEP7	14-3-3 protein epsilon	0	1	1	1	1	0	1	1	0	0
YWHAG	14-3-3 protein gamma	4	4	4	6	4	3	6	5	1	1
YWHAH	14-3-3 protein eta	0	0	1	1	1	1	2	2	0	0
YWHAQ	14-3-3 protein theta	2	3	3	2	4	2	4	4	0	1
YWHAZ	14-3-3 protein zeta/delta; isoform X1	3	2	7	7	6	6	6	7	2	2
ZFYVE21	zinc finger FYVE domain-containing protein 21	0	0	0	0	0	1	0	0	0	0
ZNF333	zinc finger protein 333; isoform X7	0	0	0	0	0	1	0	0	0	0
ZNF34	zinc finger protein 34	0	0	1	0	0	0	0	0	0	0
ZRANB2	zinc finger Ran-binding domain-containing protein 2	1	0	0	0	0	0	0	0	0	0
